# Designing optimized drug candidates with Generative Adversarial Network

**DOI:** 10.1186/s13321-022-00623-6

**Published:** 2022-06-26

**Authors:** Maryam Abbasi, Beatriz P. Santos, Tiago C. Pereira, Raul Sofia, Nelson R. C. Monteiro, Carlos J. V. Simões, Rui Brito, Bernardete Ribeiro, José L. Oliveira, Joel P. Arrais

**Affiliations:** 1 Univ Coimbra, Centre for Informatics and Systems of the University of Coimbra, Department of Informatics Engineering, Coimbra, Portugal; 2 BSIM Therapeutics, Instituto Pedro Nunes, Coimbra, Portugal; 3 IEETA, Department of Electronics, Telecommunications and Informatics, University of Aveiro, Aveiro, Portugal

**Keywords:** Drug design, SMILES, Generative Adversial Network, Multiobjective optimization, NSGA, QSAR, GAN, RNN

## Abstract

Drug design is an important area of study for pharmaceutical businesses. However, low efficacy, off-target delivery, time consumption, and high cost are challenges and can create barriers that impact this process. Deep Learning models are emerging as a promising solution to perform de novo drug design, i.e., to generate drug-like molecules tailored to specific needs. However, stereochemistry was not explicitly considered in the generated molecules, which is inevitable in targeted-oriented molecules. This paper proposes a framework based on Feedback Generative Adversarial Network (GAN) that includes optimization strategy by incorporating Encoder–Decoder, GAN, and Predictor deep models interconnected with a feedback loop. The Encoder–Decoder converts the string notations of molecules into latent space vectors, effectively creating a new type of molecular representation. At the same time, the GAN can learn and replicate the training data distribution and, therefore, generate new compounds. The feedback loop is designed to incorporate and evaluate the generated molecules according to the multiobjective desired property at every epoch of training to ensure a steady shift of the generated distribution towards the space of the targeted properties. Moreover, to develop a more precise set of molecules, we also incorporate a multiobjective optimization selection technique based on a non-dominated sorting genetic algorithm. The results demonstrate that the proposed framework can generate realistic, novel molecules that span the chemical space. The proposed Encoder–Decoder model correctly reconstructs 99% of the datasets, including stereochemical information. The model’s ability to find uncharted regions of the chemical space was successfully shown by optimizing the unbiased GAN to generate molecules with a high binding affinity to the Kappa Opioid and Adenosine $A_ {2a}$ receptor. Furthermore, the generated compounds exhibit high internal and external diversity levels 0.88 and 0.94, respectively, and uniqueness.

**Supplementary Information:**

The online version contains supplementary material available at 10.1186/s13321-022-00623-6.

## Introduction

In drug development, for a new drug to reach the final step and get approved, an estimated $2.8 billion has been spent, and between 10 and 15 years of research were necessary [1, 2]. This is due to the fact that most drug candidates fail before reaching the last step of the process, with recent estimates pointing to a success rate of only 2% [3]. Such a low success rate implies that this is not just an expensive process but a high-risk one from a financial point of view, as most investments will fail. The high dimensionality of the chemical space has been identified as one of the main challenges, since it has been estimated that between $$10^{33}$$ and $$10^{60}$$ could be synthetically accessible [4, 5] and only a small fraction of this chemical space has been explored [6]. As the evaluation of the chemical space is a prohibitively expensive process, it is crucial to find new strategies that can effectively narrow down the search space. Deep Learning (DL) methodologies have been gaining momentum as a promising solution for de novo drug design, whose goal is to generate novel molecular compounds that exhibit specific properties, such as being active towards a predefined biological target [7]. Two steps can be typically identified in this process: the first concerns creating a model that can replicate the chemical space. In contrast, the second focuses on optimizing the aforementioned model so that it is able to generate new molecules that exhibit specific properties.

Recurrent Neural Networks (RNNs) were the first DL-based technique that was successfully applied to drug generation. This type of network can learn and capture the syntax of sequences of data, which is the case of molecules, represented as Simplified Molecular Input Line Entry Strings (SMILES). Gómez-Bombarelli et al. proposed chemical VAE (CVAE), which transforms a SMILES sequence to and from fixed-sized continuous vectors [8]. In fact, a chemical Variational Autoencoder (VAE) was first used in this context by Gomez-Bombarelli et al. to transform the SMILES discrete data into a real-valued continuous vector [8]. In this work, a model that can predict specific properties from the latent vector was also trained. Gupta et al. and Segler et al. implemented a SMILES generator by using LSTM cells [9, 10]. The former also showed that its method could be applied to low-data drug discovery and fragment-growing while advocating that transfer learning avoids introducing errors or unwanted bias compared to RL. Olivecrona et al. and Liu et al. resorted to this methodology to generate new molecules. They then optimized the model with the REINFORCE algorithm [11], a policy-based Reinforcement Learning (RL) method, in order to bias it towards the space of desired properties [12, 13]. Popova et al. approached this problem by pre-training two independent networks: a stack-augmented RNN Generator and a Predictor, which are then used jointly through RL to optimize the generator. They showed that their model could be effectively biased towards physical properties like the melting temperature and partition coefficient, specific biological activity, and chemical complexity [14]. Zheng et al. trained an RNN model with GRU cells on a biogenic dataset that includes stereo-chemical information to learn the grammar of these SMILES strings with higher complexity. They then fine-tuned it by employing Transfer Learning [15].

The approaches mentioned above can suffer from exposure bias [16, 17], which prompted the appearance of other DL-based alternatives for targeted generation of compounds, mainly adversarial approaches. A Variational Autoencoder (VAE) was first used in this context by Gomez-Bombarelli et al. to transform the SMILES discrete data into a real-valued continuous vector [8]. In this work, a model that can predict specific properties from the latent vector was also trained. Blaschke et al. studied and compared VAEs and adversarial autoencoders and were able to show that both methodologies result in the preservation of the chemical similarity of the input molecules [18].

A generative adversarial network (GAN) is a particular type of neural network model where two networks are trained simultaneously, with one concentrated on image generation and the other centered on discrimination [19]. The generator generally catches the distribution of true examples for new data sample generation. The discriminator is usually a binary classifier that distinguishes between produced and true samples as accurately as feasible. An original deep neural network (DNN) architecture called a reinforced adversarial neural computer was employed for de novo drug design of novel small-molecule organic structures based on a GAN and reinforcement learning [20]. This work uses a differentiable neural computer, a category of neural network with increased generation capabilities due to the addition of an explicit memory pool mitigating typical issues found in adversarial settings, as the generator. This model generated structures that match the distributions of key chemical descriptors, and the lengths of SMILES strings in the training dataset [20]. LatentGAN is another deep-learning architecture that combines an autoencoder, and a GAN for de novo drug design [21]. Prykhodko et al. used an autoencoder to find a numerical representation of the SMILES Strings that can be used to train a Generative Adversarial Network (GAN), surpassing the differentiation problem that would arise if the discrete SMILES data were directly used [21]. Even with the augmentation technique, the proposed autoencoder could only reconstruct 82% of the molecules properly. The author also showed that Transfer Learning could optimize the generation process.

There are also applications of Graph Neural Networks in de novo drug design, such as Xiong et al.’s work that considers three aspects: molecule scoring, molecule generation, optimization, and synthesis planning [22]. Also, the author in [23] adopted Transformer to generate molecules. The network takes an amino acid sequence as input and generates molecules with the predicted capacity to bind the protein target. The model outputs valid structures with reasonable values of computed physicochemical characteristics, a drug-likeness metric, and a synthetic accessibility score.

Among proposed approaches in this topic, the RL-based approaches are the most common, but have a tendency to focus on local minima and therefore return very similar and sometimes duplicate molecules. Applying a Generative Adversarial Network (GAN) that includes an optimization strategy is an alternative approach that increases the diversity inside the generated compounds and resolves the problem of exploitation exploration of the reinforcement technique.

Moreover, stereochemistry, which is of the utmost importance in drug design and action, has not yet been appropriately considered. In fact, stereochemistry is extremely important in drug development, and introducing this information helps improve the applicability of existing models. To the best of our knowledge, the data with stereochemistry information filtered from the training dataset in the state-of-the-art, as it decreases the percentage of validly generated molecules and only has been considered by Zheng et al. [15].

Additionally, drug design is inherently a multiobjective problem where molecules must have several properties to satisfy. The compounds must represent a set of characteristics that ensure their effectiveness, selectivity, permeability, synthesizability, and solubility. However, a complete system that generates valid molecules and optimizes multiple traits has remained elusive.

The aforementioned issues are addressed in this work by proposing a framework to generate candidate drug molecules. The framework includes an autoencoder with compound SMILES embedding, feedback GAN with gradient penalty for sequence generation, and an LSTM property predictor for feedback to GAN. Moreover, we integrate a multiobjective optimization strategy based on a non-dominated sorting genetic algorithm to generate a more accurate set of molecules. The method was applied to designing inhibitors for the kappa opioid receptor (KOR) and Adenosine $A_ {2a}$ receptor (ADORA2A).

## Methods

**Fig. 1:**
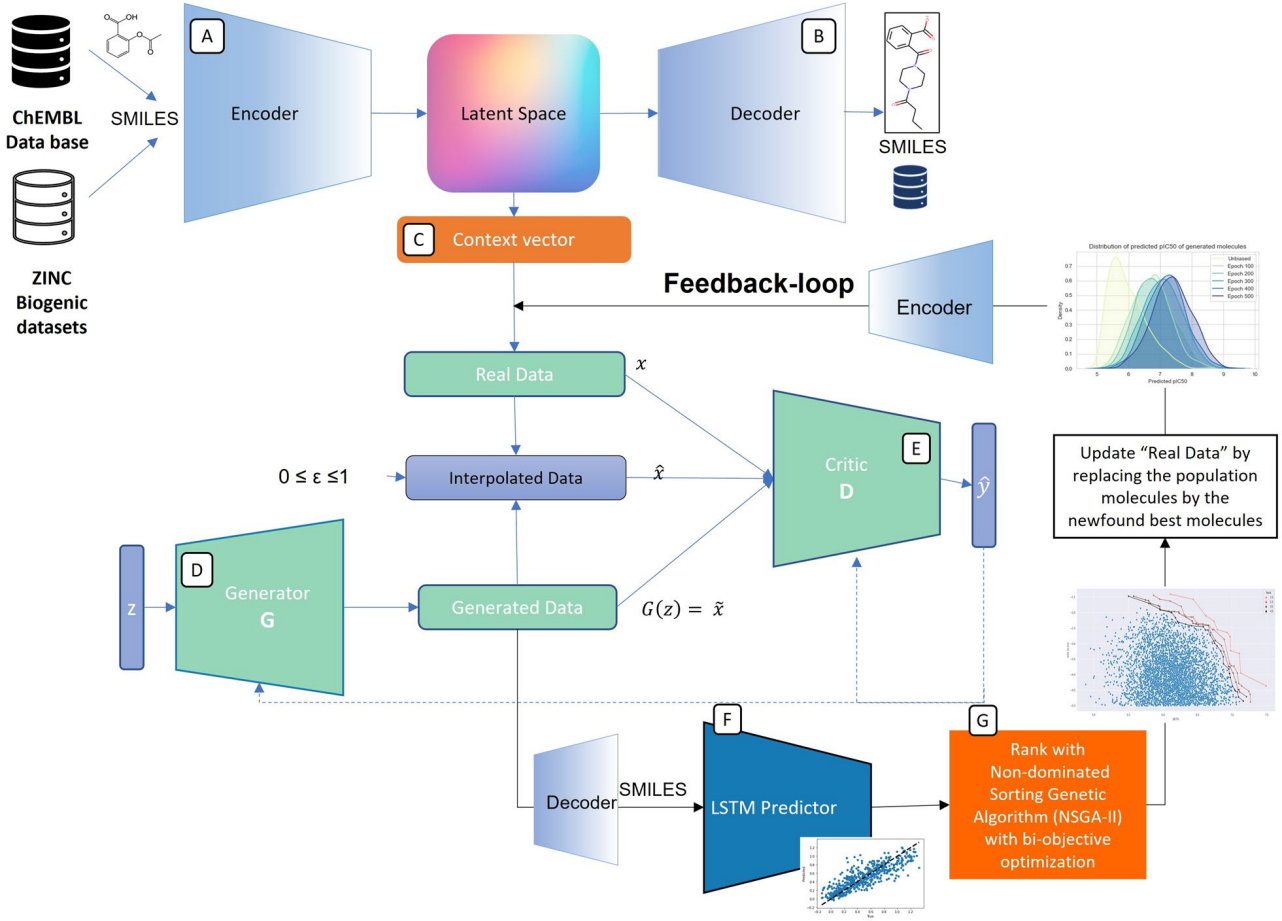
The general workflow. This model is composed of an Encoder–Decoder (**A** and** B**) that converts SMILES into latent space vectors that are then used as real data in the training of a WGAN-GP network that comprises a Generator (**D**) and Critic (**E**). The feedback-loop, Predictor (**F**), and selecting Pareto optimal molecules by NSGA-II algorithm (**G**) are only active during the optimization step

The general proposed framework is illustrated in Fig. 1 and comprises an autoencoder, more specifically, an Encoder–Decoder architecture based on RNNs [24, 25], a Wasserstein GAN with gradient penalty (WGAN-GP) [26], a Predictor and an optimization step based on feedbackGAN [27]. The Encoder–Decoder architecture allows the model to learn a context vector (Fig. 1C) which is a new fixed-length representation of SMILES strings that can be mapped back to the original molecule. Therefore, the encoder (Fig. 1A) can be used to transform an entire dataset of SMILEs into an equivalent dataset that consists of context vectors. This new equivalent dataset is used as real data to train the WGAN-GP so that, once trained, the generator (Fig. 1D) is able to generate new samples that follow the same distribution as the context vector’s dataset. In order to obtain the corresponding SMILES strings, these samples must then be passed through the decoder (Fig. 1B). By combining a GAN with an autoencoder, it becomes possible to train the WGAN-GP, surpassing the differentiation problem associated with discrete data, such as SMILES strings [28]. In this manner, the generator is able to generate molecules that span the entire chemical space. However, in drug design, the goal is to generate compounds that exhibit multiple desired properties. In order to do so, an optimization step based on feedbackGAN [29] was devised that slowly forces the generator to focus on specific regions of the chemical space. Unlike the previous steps, this is a goal-specific step, as it requires Predictor Module (Fig. 1F) for the desired properties that can identify interesting compounds and select a set of Pareto front molecules with non-dominated score vectors (Fig. 1G). These compounds are then included in the training data. This leads to a slow but effective shift in the generated distribution towards the desired region of the chemical space. In this step, the best of the generated molecules are selected by the novel application of a non-dominated sorting algorithm, a proven multiobjective optimization method. We optimize different criteria of drug candidates such as the binding affinity *pIC*50, topological polar surface area (TPSA) [30] of a molecule, the partition coefficient, the solubility (LogP) and synthetic accessibility score (SAS) [31].

### Datasets

To train the Encoder–Decoder and the WGAN-GP, a dataset containing SMILES strings from the ChEMBL [32] and Zinc Biogenic [15] databases is used, including, molecules with and without stereo-chemical information containing the corresponding tokens.

For training and testing the predictor module, we used the Kappa Opioid Receptor (KOR), $A_ {2a}$ receptor (ADORA2A), Ubiquitin specific protease 7 (USP7) [33] and Tyrosine-protein kinase JAK2 (commonly called JAK2) and their corresponding *pIC*50 values.

Table 1 shows the detailed information of the datasets that been used throughout this article, which are available at https://github.com/larngroup/GAN-Drug-Generator. Once the best architecture and set of hyperparameters had been defined, the general model was trained on two other more complex datasets: composed_dataset_1 and composed_dataset_2 which contain 100,000 and 500,000 drug-like molecules, respectively, that were retrieved from the datasets mentioned in Table 1. This resulted in datasets that include a wider variety of compounds and molecules with and without stereochemistry.

**Table 1:** Summary of the datasets used throughout the experiments

Dataset	# Compounds	Labeled	Observations
ChEMBL [32]	1,178,946	No	
Zinc Biogenic [15]	108,283	No	
ADORA2A	4729	Yes	CHEMBL251
KOR	5262	Yes	CHEMBL237
JAK2	1697	Yes	CHEMBL2971
USP7 [33]	1109	Yes	CHEMBL2157850
bbbp [27]	1340	Yes	
composed_dataset_1	100,000	No	
composed_dataset_2	500,000	No	

### Data preprocess

Stereochemistry is the study of stereoisomers, which are molecules with the same chemical formula and bound atom sequence but differ in the three-dimensional orientations of their atomic arrangement. Isomers have different geometries and therefore interact differently with the surrounding environment. A famous example of this phenomenon is Iboprufen, where only one of the isomers of the active substance (sold as a mixture) is, in fact, active. Sometimes, however, two or more stereoisomers are very hard or even impossible to isolate or are interchangeable, and stereochemistry is generally omitted for these. But this is not the case for carbon stereocenters, which is the problem we address here. Molecules with stereochemistry information include chiral centers, charges, and cyclic connections, which are shown in SMILES notation with characters such as ‘$+$’, ‘−’, ‘@’, ‘/’ or ‘$\backslash$’, etc [34]. Moreover, the SMILES format has a form of depicting stereochemistry around each stereocenter. In double bonds, the characters ‘/’ and ‘$\backslash$’ will denote orientation around it and only make sense if pairs (for example, ‘$/C=C/$’) denote a cis and ‘$\backslash C=C/$’ a trans configuration). In chiral centers, the characters ‘@’ or ‘@@’ depict the local configuration of the ligands. However, they have no direct correspondence to R and S isomers until we consider the order of appearance of atoms in the SMILES string. This system, although redundant, is not ambiguous in Canonical SMILES notation. Conventionally, each character in a molecular SMILES Strings is used as a token in the deep model. This technique is unable to capture the characteristics of chiral centers, charges, and other chiral properties. To preserve these features, we tokenize the SMILES containing regular tokens and combined tokens, meaning that we consider sections of the SMILES string that are enclosed in brackets (for example, ‘[C@@H]’ and ‘[N+]’) as a single token, which results in an extended vocabulary.

**Fig. 2:**
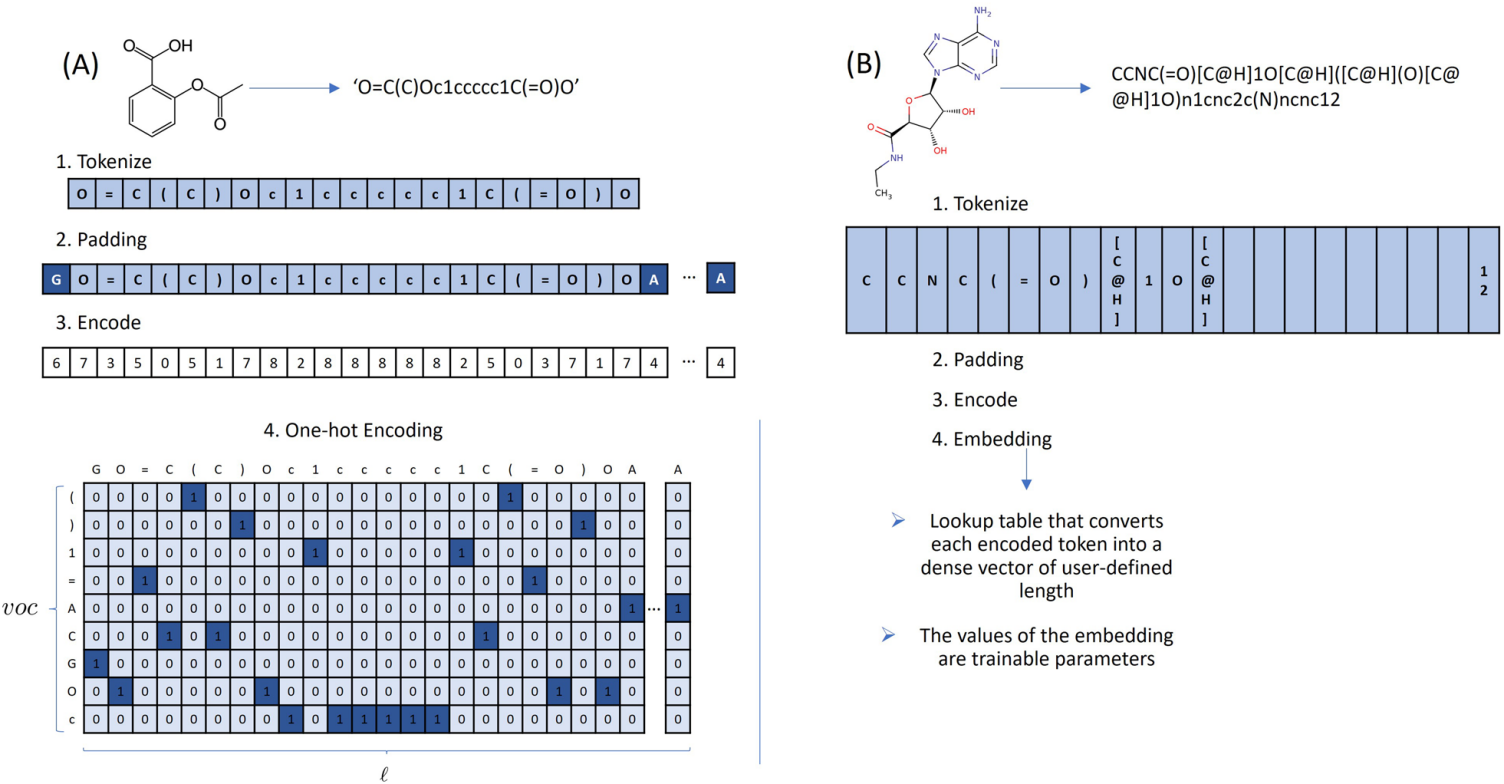
Data preprocessing of the SMILES string.** A** Acetylsalicylic Acid using One-hot Encoding and** B** a sample Adenosine Receptor BDBM21220 through embedding method

Therefore, the SMILES strings are canonicalized, checked for duplicates, and then preprocessed. The preprocessing step consists of the above tokenization followed by adding ‘G’ at the beginning of each SMILES string and ‘A’ at the end and padding. The tokenized SMILES are then either One-hot Encoded (OHE), where each token becomes a binary vector, or passed through an embedding layer that converts each token into a dense vector. Fig. 2 shows this process in more detail.

### Encoder–decoder model

The Encoder–decoder model, also known as a sequence to sequence model, is an autoencoder that contains recurrent layers, which allows it to work with sequences of data [35]. This type of model is typically used when the goal is to predict a new sequence of words that is in some way related to the input sequence, such as language translation tasks. The Encoder–Decoder learns a new representation of the input data, denoted by the context vector. This work uses this architecture to find a new continuous representation of SMILES strings that can then be used as training data for a GAN model, overcoming the inability to backpropagate through discrete data that arises if a GAN is trained directly on the SMILES String.

The encoder translates the input SMILES string into a context vector belonging to the latent space while the decoder, given this context vector, reconstructs the initial SMILES Strings. Therefore, the model is trained using the categorical cross-entropy between the input SMILES and the predicted SMILES as the loss function. The optimizer uses the gradient of this loss function to update the network’s weights.

**Fig. 3:**
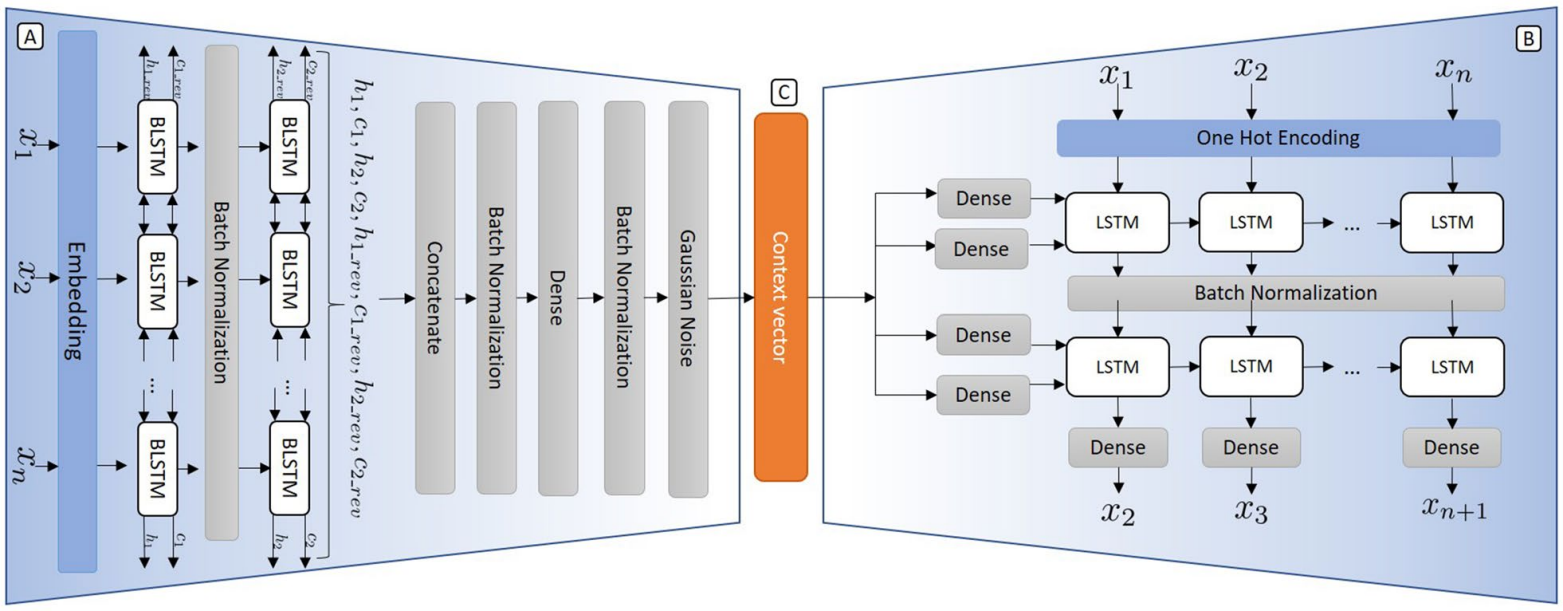
The detailed structure of the Encoder (**A**) and Decoder (**B**). This model is used to convert the SMILES strings into vectors in the latent space [context vector (**C**)]

The proposed Encoder–Decoder model is represented in Fig. 3 resulted from the evaluation of several architectures and numerous hyperparameters. The Additional file 1: Section 2 contains a summary of these parameters and a detailed study of the efficiency of the model through grid search strategy. The optimized model consists of an encoder module with an embedding layer as its input layer, followed by two bidirectional LSTM layers with 512 units. The final hidden and cell states are retrieved from the bidirectional layers and concatenated before going through a dense layer (with as many units as the desired length of the context vector, 256 in this case) and a Gaussian Noise layer (standard deviation of 0.1). This last layer is only active during training and allows the context vector to become more robust. The decoder uses the context vector to feed four independent dense layers tasked with creating the hidden and cell states that will be given as initial states to its two stacked LSTM layers (with 512 units) followed by a dense layer. This dense layer includes a *softmax* activation layer in order to output the probabilities associated with the next token. Also, in between every one of the aforementioned layers, in both the encoder and decoder, a batch normalization layer is included (with batch normalization momentum of 0.9), which speeds up training and reduces overfitting by normalizing the output of the previous layer [36].

### Wasserstein GAN with Gradient-penalty

We adopted Wasserstein GAN with Gradient-penalty (WGAN-GP) since this type of model exhibits increased performance and stability [26] when compared to the traditional GAN as proposed by Goodfellow et al. [28]. GANs cannot be directly applied to categorical data, like SMILES strings, due to the fact that the sampling process at the end of the generator does not allow for the back propagation of the errors through that layer. However, since the proposed Encoder–Decoder model can find an alternative continuous representation for the SMILES strings, this new latent space representation can be used as real data to train the WGAN-GP without requiring intrinsic changes to the model. As the training data now consists of vectors, both the Critic and the Generator can be simple Feed Forward Neural Networks. These two neural networks have opposing objectives, with the critic aiming at distinguishing between generated $\tilde{x}$ and real data *x*, and the generator trying to fool the critic into believing that its data is real. A gradient penalty (weighted by $\lambda$) is included in the loss function (*L*) to prevent the critic’s gradient from deviating from 1. Equation 1 shows this loss function, where the third term corresponds to the gradient penalty.

1 $$\begin{aligned} L= {\mathbb {E}}_{\pmb {z}\sim p_{\pmb {z}}} \left[ D(G(\pmb {z})) \right] -{\mathbb {E}}_{\pmb {x}\sim p_{data}} \left[ D(\pmb {x})\right] \nonumber \\& + \lambda {\mathbb {E}}_{\pmb {\hat{x}} \sim p_{\pmb {\hat{x}}}} \left[ \left( \Vert \nabla _{\pmb {\hat{x}}} D(\pmb {\hat{x}}) \Vert _{2} -1 \right) ^{2} \right] \nonumber \\ \text{ If } \quad \tilde{\pmb {x}}= G(\pmb {z}) \quad \text{ then } \quad \hat{\pmb {x}} = \epsilon * \tilde{\pmb {x}} + (1-\epsilon )*\pmb {x} \quad \text{ with } 0 \le \epsilon \le 1 \end{aligned}$$

where $\epsilon$ is uniformly sampled between 0 and 1. In practice, the gradient penalty amounts to creating a set of interpolated samples $\hat{\pmb {x}}$ (each using a real sample $\pmb {x}$, a generated sample $\tilde{\pmb {x}}$ and $\epsilon$) that result from randomly choosing points that lie on the lines that connect the batch of real samples to the batch of fake samples and evaluating its gradients. The gradient penalty loss term returns the squared distance between the gradient calculated at the interpolated points and 1. Therefore, it penalizes the critic whenever its gradient deviates from 1, thus enforcing the 1-Lipschitz constraint [26].

The implemented WGAN-GP model includes a critic network and a generator network. The critic contains two dense layers with 256 units each and Leaky-Relu as the activation function (with $\alpha = 0.3$) and a final dense layer (256 units) with no activation function. The generator comprises five dense layers with Leaky-Relu activation function ($\alpha = 0.3$): the first contains 128 units and the remaining layers contain 256 units each; a batch normalization (momentum of 0.9) is included between the aforementioned layers. The input to the generator is a 64 dimensions vector drawn from the uniform distribution. Both networks are trained using the Adam optimizer with a learning rate of 0.0001 and drop-out value of 0.2.

### Case-study

In this study, we used Kappa Opioid Receptor (KOR) and $A_ {2a}$ receptor (ADORA2A) as case studies. The Kappa Opioid Receptor is one of four opioid receptors that belong to the G-protein-coupled receptors (GPCR) superfamily. Research on opioid receptors, particularly on KOR, has been gaining momentum as it mediates affective disorders such as depression and anxiety, neurological diseases like epilepsy, but also pain and drug addiction, making it a promising pharmacological target [37–40]. The ADORA2A is also a part of the GPCR superfamily and mediates pain, motor control, and mood [40]. The interest in ADORA2A, present in the central nervous, stems from the fact that it plays a crucial role in modulating motor functions, maintaining a low level of non-motor side effects. Therefore, ADORA2A antagonists are an attractive non-dopaminergic option for treating Parkinson’s disease. The goal of the current experiment is to find ligands that bind to the KOR/ADORA2A and blocks their biological responses. To attain it, the generator of the WGAN-GP model must be optimized to generate compounds with a high affinity to bind antagonistically. The *pIC*50 is used to measure this, which is the negative logarithm of the half-maximal inhibitory concentration. Thus, the higher the *pIC*50, the more potent the inhibition.

### Predictor model

**Fig. 4:**
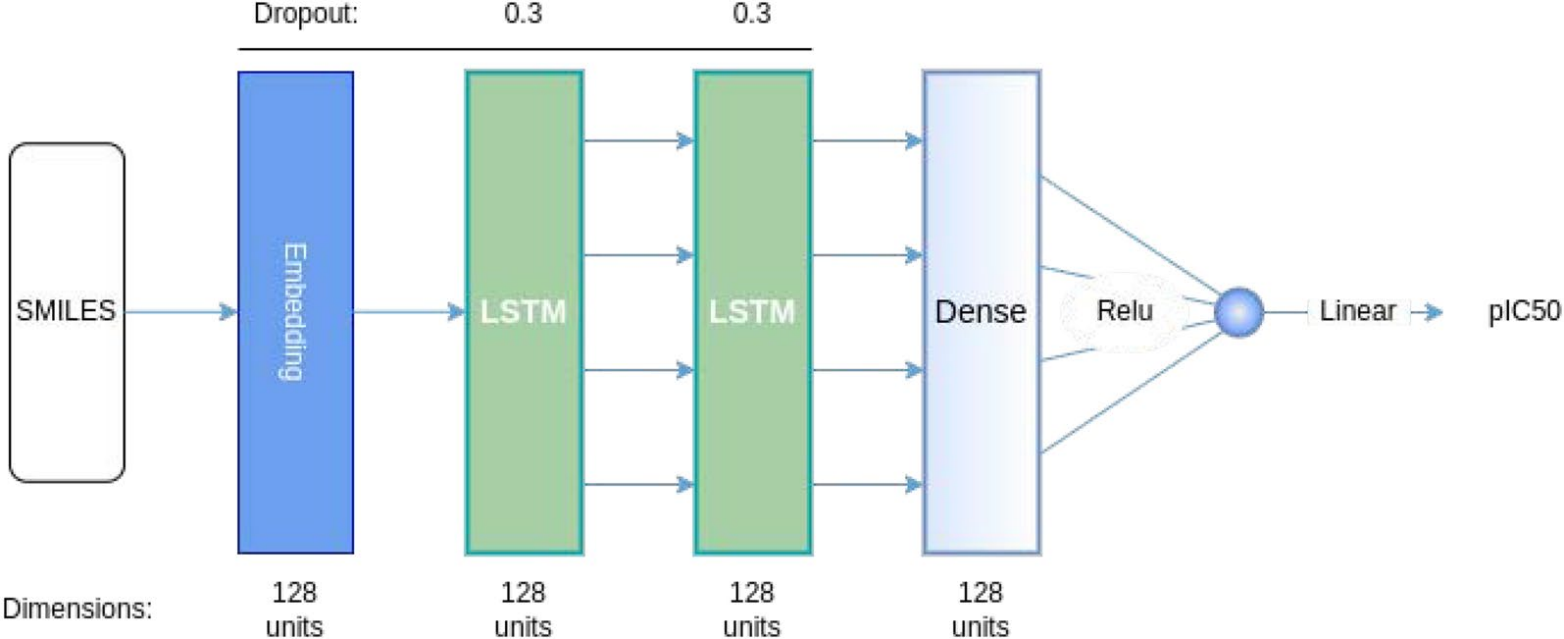
General schema of LSTM-based Predictor architecture. This regression model aims to predict the affinity of a given molecule in the format of SMILES string

In order to evaluate the optimization process, a QSAR model, henceforth denominated by Predictor, was implemented [41]. This regression model aims to predict the affinity of a given molecule as measured by the pIC50. The Predictor is an LSTM-based model that receives tokenized and padded SMILES strings as input, which are then passed through an embedding layer followed by two LSTM layers and two dense layers. Since this is a regression problem, the last layer has a single unit and a linear activation function. The use of RNN-based QSAR models is of particular interest for two reasons: first, this type of model works with inputs with different lengths, and second, it also works with SMILES strings, which means that there is no need to find other types of molecular representations that might add human bias [42]. The optimal architecture for the Predictor consists of an embedding layer with 128 units, followed by two LSTM layers and a dense layer (128 units each), and a final layer with a single unit (Fig. 4).

### Optimization through FeedbackGAN

After training the implemented WGAN-GP model, the Generator is able to produce context vectors that are then decoded into SMILES strings that span the chemical space. In order to optimize the proposed framework, a feedbackGAN based approach is devised to bias the model toward specific properties. FeedbackGAN is an optimization framework proposed by Gupta et al. [29] that resorts to a feedback-loop and a function analyzer to optimize a GAN towards the space of desirable properties. In the context of the current problem, the Predictor takes the place of the function analyzer for binding affinity score. Moreover, we use the RDkit tools [43] to calculate other properties of the generated candidate molecules, such as logP, TPSA. We generate python scripts to calculate the SAS. After training the WGAN-GP model, the Predictor is linked to the GAN through a feedback mechanism. The GAN then enters a retraining phase in which, at the end of each epoch, the Generator is used to sample a set of new molecules fed to the Predictor, or RDKIT functions to be evaluated. At each epoch, the *n* training set of SMILES with the dominated scores are replaced by the *n* non-dominated score newly generated SMILES. By doing so, the training data is constantly being updated with new and better molecules according to the optimization score vector that has been considered. This results in a fine-tuned Generator that gradually approaches the space of the desired property.

### Multiobjective molecular selection

We applied the non-dominated sorting algorithm to select the best-generated molecules. The goal is to apply a more thorough assessment to each molecule by analyzing the influence of different objectives to be optimized. This analysis will be integrated into a Feedback dynamic so that the Generator can learn from its experience. It is necessary to obtain a promising set of lead molecules that consider different molecular properties simultaneously. These properties will include the biological affinity for the target, topological polar surface area of a molecule, the partition coefficient, the solubility (LogP), and the synthetic accessibility score of the generated compounds. This optimization step is carried out based on the most common multiobjective formalism for evaluating the molecule as a whole in multiobjective problems, as indicated in the following (vector) score functions:

#### Definition 1

Let *m* be a generated molecule. Let *PIC*50(*m*) and *SAS*(*m*) indicate the binding affinity score and synthetic accessibility score respectively in the generated molecule *m* and let *TPSA*(*m*) and *LogP*(*m*) indicate the topological polar surface area (TPSA) and logP score, respectively. The following (vector) score functions can be defined:

$$\begin{aligned}&\left( P_{IC50}(m), -SAS(m)\right) \\&\left( P_{IC50}(m), -TPSA(m)\right) \\&\left( -LogP(m), -SAS(m)\right) \\ \end{aligned}$$

Note that this formulation can be extended to any other molecular properties. At last, to select from the pool of compounds with maximized properties, we intend to apply the Pareto ranking techniques. This work employs the Fast Non-dominated Sorting Genetic Algorithm (NSGA-II) [44] to compare generated molecules and evaluate them based on the criteria outlined. Each generated molecule is ranked based on the number of molecules in the population that it is dominated by. Dominated molecules are given values between 1 and $P-1$, where *P* are the total number of generated molecules in the generated population at each step, corresponding to how many other molecules they are inferior to. This algorithm was chosen as a ranking method because of its simplicity and efficiency, and computational complexity $O(n^2)$. The Additional file 1: Section 4 provides a more detailed explanation of this algorithm.

### Validation strategy

The validation strategy for the Encoder–Decoder model is based on the percentage of molecules that it can correctly reconstruct. As the training dataset only contains valid molecules, a correctly reconstructed (CR) SMILES string is automatically valid. The SMILES generated by the framework are syntactically and biochemically validated by RDkit tools [43], and also by four other metrics inspired by the Guacamole framework [45], which are the following: the percentage of valid molecules (Validity), the percentage of uniquely generated molecules (Uniqueness), i.e. non-repeating SMILES, the percentage of generated molecules that don’t appear on a reference dataset (Novelty), and finally the Kullback-Leibler (KL) divergence between datasets. The KL divergence uses several properties of the molecules also calculated using the RDkit tools, namely: NumRotatableBonds, MolLogP, MolWt, BertzCT, TPSA, NumHAcceptors, MolWtNumHDonors, NumAliphaticRings, and NumAromaticRings. Besides these properties, it is also included the maximum nearest neighbor using ECFP4 fingerprints. Specifically, the KL divergence is calculated as presented by the following equation:

2 $$\begin{aligned} S = \frac{1}{k} \sum _{i}^{k} exp \left( -D_{KL,i}\right) \end{aligned}$$

where k is the number of calculated features, and $D_{KL,i}$ is the KL divergence calculated between the datasets of the *i* property’s value distribution.

Finally, the *Internal Diversity* (Int Div) and *External Diversity* (Ext Div) are also computed. The diversity is evaluated by resorting to the Tanimoto Similarity, which computes the similarity between two molecules in terms of their circular fingerprints [46]. The Tanimoto distance can be defined as $1-T_{s}$. From it, the diversity between two sets of generated molecules *A* and *B* can be defined as the average of the Tanimoto distance between every single pair of molecules:

3 $$\begin{aligned} Div(A,B) = \frac{1}{|A|\cdot |B|} \sum _{a\in A}^{|A|} \sum _{b\in B}^{|B|} \left( 1-T_{s}\right) \end{aligned}$$

Therefore, the internal diversity is obtained by simply computing *Div*(*A*, *A*) and the external diversity by *Div*(*A*, *B*), where *A* is the set of generated SMILES and *B* is the set of the training data.

## Results and discussion

### Experimental analysis on the encoder–decoder

The Encoder–Decoder model is trained using a batch size of 128 and the Adam optimizer (learning rate of 0.01). The maximum number of epochs is set to 100, but only the models with the best validation loss are considered (10 % of the data is used to validate the model). During the training phase, the teacher’s forcing algorithm is applied so that the ground truth of the current step is given as input to the following step in order to minimize the propagation of errors. Table 2 shows the results obtained when training the aforementioned model with 500,000 SMILES Strings. It can be observed that the model effectively learned to map SMILES strings to the latent space and vice-versa, with a percentage of correctly reconstructed molecules of 99.2% and 99.0% for the training and test data set, respectively. It is important to note that correctly reconstructed (CR) molecules are automatically valid and that the validity is constantly higher, meaning that some molecules are reconstructed into valid molecules but not the same molecules that are given as input.

**Table 2:** Performance of the Encoder Decoder model based on percentage of Correctly Reconstructed (CR) molecules and validity of the train and test sets

% CR (train)	% CR (test)	% Validity (train)	Validity (test)
99.2	99.0	99.9	99.8

### Experimental analysis on WGAN-GP

**Fig. 5:**
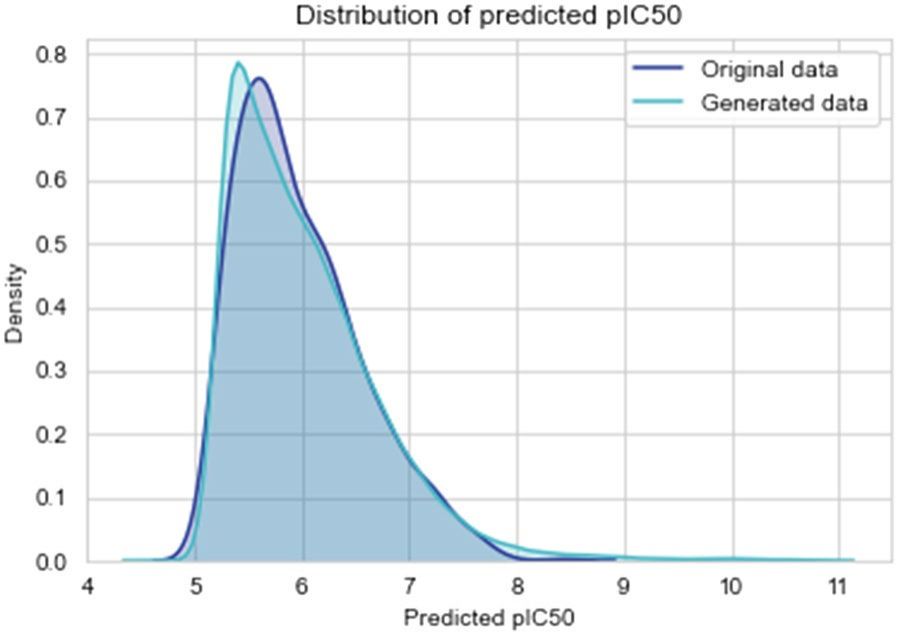
Comparison of the predicted *pIC* 50 distributions for the original data and generated data by WGAN-GP model

The implemented WGAN-GP model is trained on 100,000 molecules and for 10,000 epochs. The results of sampling 1,000 valid molecules using the whole framework are presented in Table 3 and Fig. 5 from which it is possible to conclude that the GAN effectively learns the training data distribution as it generates data that follows that same distribution regarding the values of predicted *pIC*50. It is worth noting that the validity of the generated data is only 30.2%. This low value is attributed to the fact that the WGAN-GP is learning to mimic a distribution of continuous vectors that are then converted into a series of discrete tokens that need to abide by certain rules to be considered valid SMILES strings, hence the difficulty in generating valid molecules.

**Table 3:** The performance of the WGAN GP model in comparison of the original data with the generated ones

	Original data	Generated data
Maximum	9.974	8.454
Mean	6.003	5.984
Minimum	4.548	5.083
Standard Deviation	0.684	0.577
External Diversity	–	0.890
Internal Diversity	–	0.887
% Unique	–	100.0
% Valid	–	30.2

**Fig. 6:**
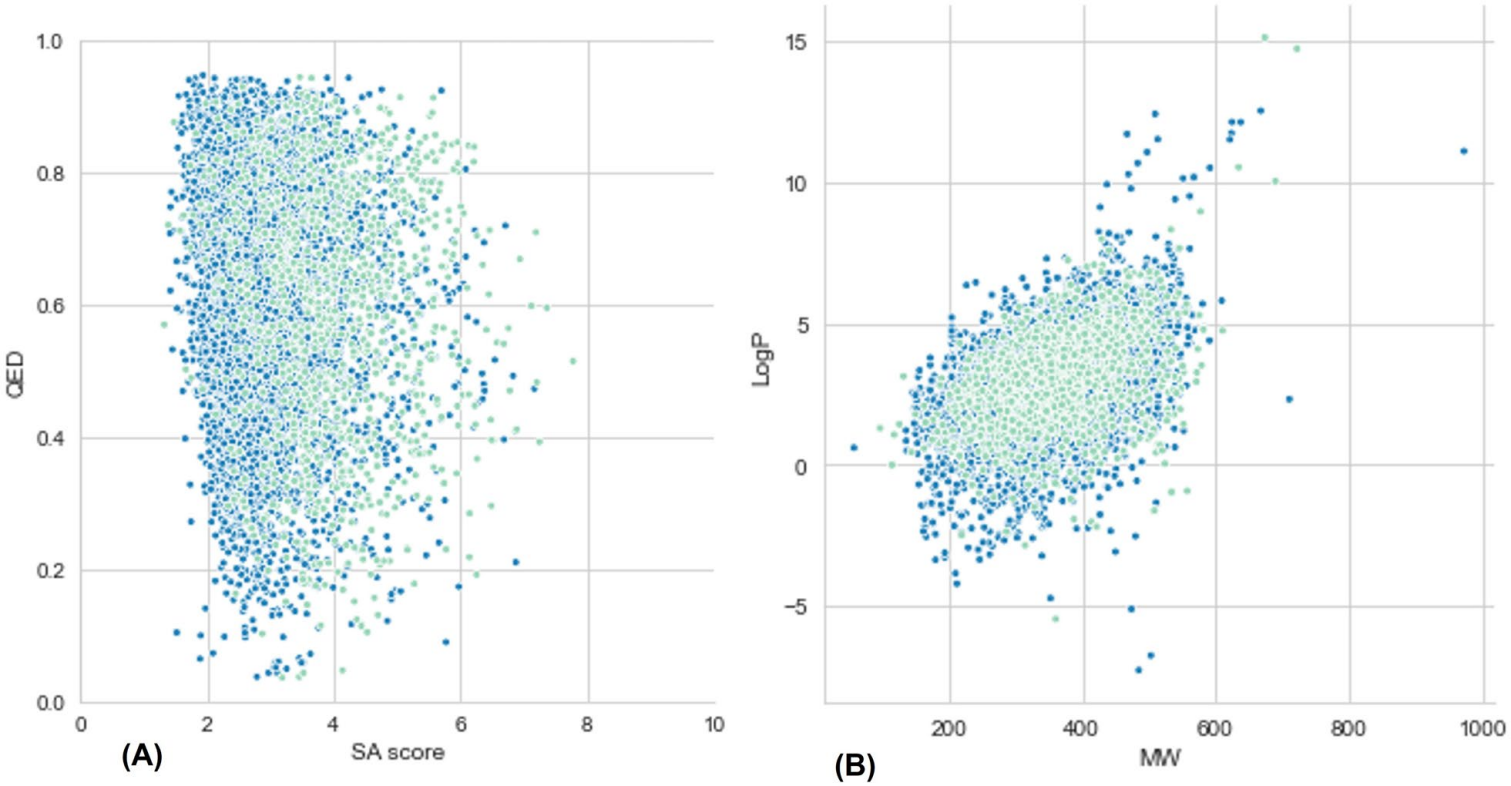
Evaluation of WGAN-GP model for the original training data and generated. **A** Evaluation of the QED and SAS.** B** Evaluation of the $\log P$ and MW

To further compare the generated data to the original training data in terms of general drug-like properties, Fig. 6 show the relationship between the Quantitative Estimate of Drug-Likeness (QED) and Synthetic Accessibility (SA) score, and the relationship between the logarithm of the partition coefficient between n-octanol and water ($\log P$) and Molecular Weight (MW), respectively. By interpreting them, it is possible to conclude that the original and generated data are clearly overlapping, which means that the GAN successfully learned the training data distribution in terms of several of its properties. It should be noted that to make the plots more perceptive, instead of resorting to the 100,000 molecules in the training data, only 5,000 were used, but care was taken to make sure that the distribution of this smaller set in terms of predicted *pIC*50 was kept the same.

### Performance analysis of the predictor

**Fig. 7:**
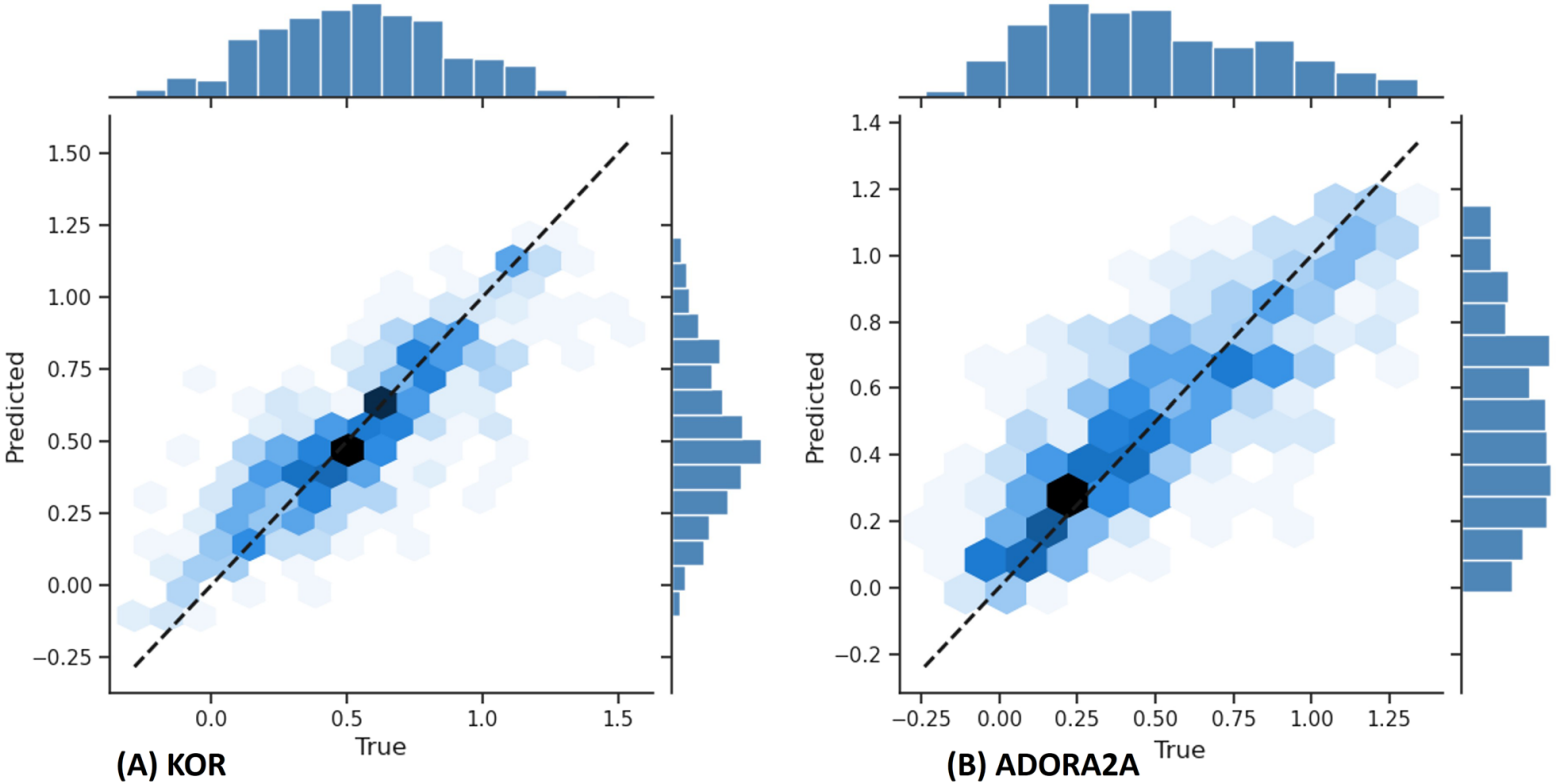
Scatter plots from applying the Predictor for the binding affinity. The plot shows the predicted *pIC*50 with the model versus true *pIC*50 and the regression line for the test set to different datasets

The Predictor module is trained to predict the binding affinity. During training, the Adam optimizer (with $\beta _1 = 0.9$ and $\beta _2 = 0.999$) with a learning rate of 0.0001 and a batch size of 16 is applied. The maximum number of epochs is set to 100, but early stopping is employed, and all the models stop training before reaching the 100th epoch. Figure 7 shows the scatter plot that results from applying the predictor to two different hold-out test sets, where it can be observed that the model performs constantly well for the complete range of values. Moreover, the Predictor module was assessed by acquiring the Mean Squared Error (MSE), Coefficient of Determination (CD), the Concordance Correlation Coefficient (CCC), and Mathews Correlation Coefficient (MCC) on different datasets: USP7, JAK2, ADORA2A, and KOR. In the training phase, five validation splits were used, and a model was created for each and independently trained. In the testing phase, each model tried to predict the test dataset and a mean of the results was calculated, as shown in (Table 4).

**Table 4:** Performance of the Predictor module on the USP7, JAK2, ADORA2A and KOR datasets

Dataset	MSE ± Std	CD ± Std	CCC ± Std	MCC ± Std
USP7	0.08 ± 0.177	0.492 ± 0.122	0.664 ± 0.088	0.736 ± 0.042
JAK2	0.063 ± 0.003	0.435 ± 0.018	0.658 ± 0.021	0.43 ± 0.042
ADORA2A	0.041 ± 0.003	0.588 ± 0.041	0.78 ± 0.02	0.586 ± 0.013
KOR	0.043 ± 0.002	0.629 ± 0.015	0.795 ± 0.007	0.664 ± 0.01

It shows the acquired values of Mean Squared Error (MSE), Coefficient of Determination (CD), Concordance Correlation Coefficient (CCC), and Mathews Correlation Coefficient (MCC), alongside their respective standard deviations (Std)

### Optimization with feedbackGAN

**Fig. 8:**
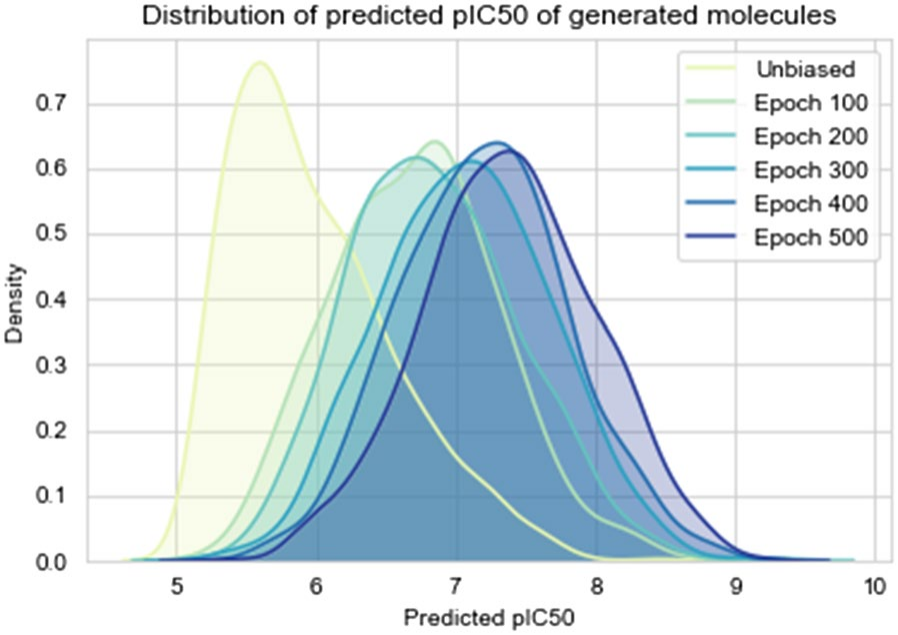
Distribution of generated molecules and the predicted *pIC* 50. The plot shows the distribution of the predicted *pIC*50 values for the unbiased model and the biased model (feedbackGAN) at every 100 epochs from the KOR dataset

The optimization has been done through feedbackGAN by maximizing KOR biding affinity. This process constantly updates the training data by replacing the worst scoring molecules with the best, which results in a gradual shift of the distribution towards the desired property. At the end of each epoch, 200 valid molecules are sampled, and 10% of the best replace the worst molecules in the “real data” training set. In order for the changes in the dataset to be significant and effectively bias the distribution that is being learned, only 5000 molecules are used as “real data” for the optimization process. These 5000 molecules are sampled from the dataset used to train the WGAN-GP, taking into consideration the distribution of the original dataset in terms of *pIC*50. The unbiased WGAN-GP model is optimized for 500 epochs, considering that the best scoring molecules are the ones with the highest *pIC*50. 1000 valid molecules are sampled in intervals of 50 epochs and evaluated in terms of distribution of predicted *pIC*50, validity, uniqueness, and diversity. These results are presented in Table 5. Figure 8 shows the distribution of predicted *pIC*50 values in terms of a probability density for intervals of 100 epochs of optimization, where it can be observed that the implemented strategy results in a successive shift of the overall distribution towards higher values of predicted *pIC*50. The same conclusion can be drawn from Table 5 where it is clear that the mean of the distributions is constantly increasing from 5.984 for the unbiased model to 7.283 for the model optimized for 500 epochs. It is important to note that the oscillations in the minimum and maximum values of the distributions are expected, as sampling is a stochastic process.

**Fig. 9:**
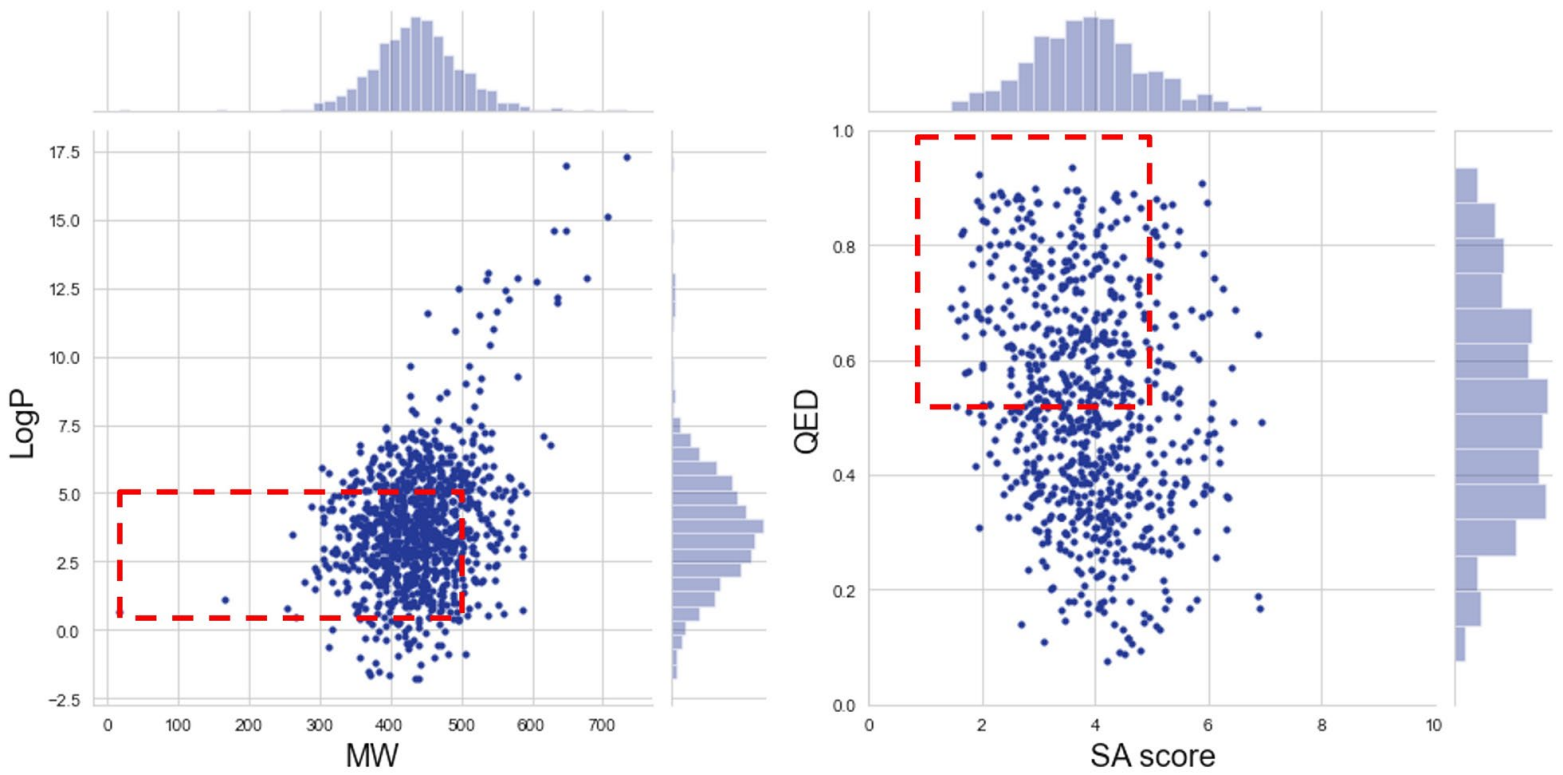
Evaluation of the $\log P$ versos MW (left) and the QED versos SAS (right) for the biased model (feedbackGAN) at 500 epochs

Although, as it has been shown, the proposed model was successful in maximizing the overall KOR affinity of the generated molecules, for them to be considered as potential drugs, it is also important to evaluate metrics such as QED, $\log P$, MW and SAS. Figure 9 shows the scatter plots and distributions of these properties for 1000 molecules generated after 500 epochs of optimization; the red square represents the region of drug-like properties. From Fig. 9 (left plot) it can be concluded that most of the molecules are outside the desired region (high QED and low SAS), with most of them exhibiting a SAS higher than 5, which implies that they are difficult to synthesize. It can be observed from Fig. 9 (right plot) that a significant amount of the generated molecules have good values for both $\log P$ and MW, even though there is a general tendency towards higher molecular weights (see Additional file 1: Section 3).

**Table 5:** Comparison of *pIC*50 distribution measures throughout the optimization process (maximization of KOR affinity). Max, Mean and Min value of *pIC*50 are shown in 500 epochs and for the unbiased (ub) model

	ub	50	100	150	200	250	300	350	400	450	500
Max	8.454	8.341	8.852	9.340	9.354	8.911	9.149	9.000	8.939	9.241	9.179
Mean	5.984	6.501	6.719	6.791	6.851	7.015	7.074	7.168	7.204	7.273	**7.383**
Min	5.083	5.198	5.205	5.380	5.181	5.496	5.271	5.340	5.522	5.380	5.380
Int Div	0.887	0.890	0.890	0.887	0.891	0.886	0.885	0.885	0.883	0.878	0.877
Ext Div	0.890	0.900	0.909	0.908	0.915	0.923	0.922	0.926	0.930	0.937	**0.938**
% Uni.	100.0	100.0	100.0	100.0	100.0	100.0	99.9	100.0	100.0	99.8	100.0
% Valid	62.2	47.1	57.7	64.0	70.7	76.0	78.9	93.6	84.1	92.4	**94.3**

### Experimental analysis on performance

**Fig. 10:**
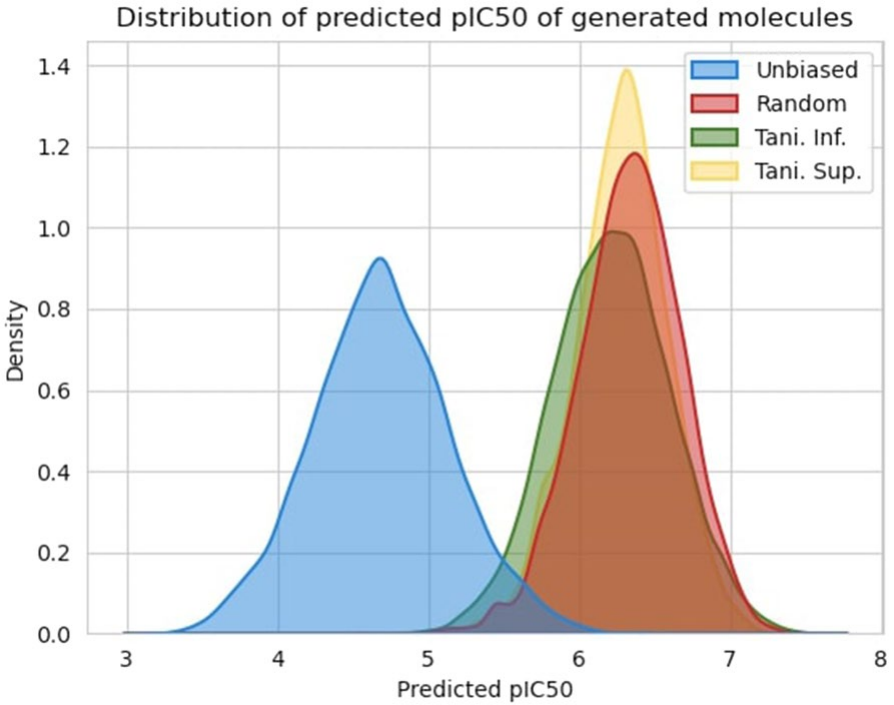
Distribution of the predicted *pIC*50 values for different sampling methods from the ADORA2A dataset

We assess the performance of the model by analyzing the feedbackGAN mechanism’s behavior given different molecule distributions as training. For the following experimental analysis, the ADORA2A molecules dataset was used. The pipeline generates the molecules based on two factors: the applied goals (i.e., for the selection of “best” molecules) and the input training dataset of the feedbackGAN. From the ADORA2A dataset, three different ways of sampling 1000 molecules were performed, named: Random, Tani-Inf, and Tani-Sup^1^. Random sampling, as the name implies, selects molecules randomly. Tani-Inf and Tani-Sup selected the molecules with the highest and lowest internal diversity of the ADORA2A dataset, respectively. The internal diversity of each molecule was calculated using the Eq. 3. In order to avoid obtaining a final sampled *pIC*50 distribution too different from the original dataset, the molecules were sampled from arbitrary *pIC*50 intervals. Then the feedbackGAN was trained for 500 epochs for each sampled data and subsequently generated, 10000 valid molecules. From Fig. 10 it can be observed that, independently of the sampling method, the generated distributions of molecules all by a moderate extent converged to the same *pIC*50 values, but with different densities. In the case of the Tani-Inf, one potential hypothesis that could explain why the molecules are spread more sparsely is that the feedbackGAN might have difficulty generating molecules similar to sampled data (i.e., the 1000 sampled data), given that the molecules are very dissimilar between themselves. From this perspective, it makes sense that the sampling Tani-Sup generates molecules less sparse, since the initial sampled data had lower internal diversity between molecules. Furthermore, the unbiased sampling of molecules (i.e., the random sampling) generates molecules with a distribution that falls between the other sampling methods (sparsity). It is important to highlight that *pIC*50 values of each molecule were calculated utilizing the trained predictor; therefore, the error might increase the further away the molecules are from the original dataset, which could also potentially explain the difference between distributions.

The molecules generated by each sampling approach are then evaluated by calculating the percentage of valid molecules (validity), the percentage of uniquely generated molecules (uniqueness), the percentage of generated molecules that do not exist in the reference dataset (ADORA2A dataset) (novelty), and finally the Kullback-Leibler (KL) divergence between the generated molecules and the reference dataset, using several molecule descriptors, as implemented in the Guacamole benchmark [45]. The latter evaluation metric consists of calculating, for both datasets (i.e., the ADORA2A and generated datasets), several descriptors from the RDKIT library, namely: NumRotatableBonds, MolLogP, MolWt, BertzCT, TPSA, NumHAcceptors, MolWtNumHDonors, NumAliphaticRings, and NumAromaticRings. On ECFP4 fingerprints, we also compute the distribution of maximal closest neighbor similarities. Each distribution of obtained values is compared between datasets using the KL divergence method, and a mean is calculated. The obtained values are presented in Table 6.

**Table 6:** Performance analysis of generated molecules considering the sampling approach of the training dataset

	Random	Tani-Inf	Tani-Sup
Validity (%)	82.81	89.89	93.22
Uniqueness (%)	96.89	94.73	97.63
Novelty (%)	100.0	100.0	100.0
KL Divergence	0.5266	0.2643	0.4915

Note that both the Tani-Inf and Tani-Sup sampling methods generate (through the feedbackGan) a higher percentage of valid molecules than Random sampling. One possible explanation can be that inputting a very dissimilar (or similar) set of molecules can “force” the model to biased generate molecules in specific “drug spaces”, while the Random sampling method gives the model an unbiased exploration direction, which could explain why it has the lowest percentage of valid molecules. The KL divergence metric corroborates to a moderate extent our premise that different sampling methods over the original dataset can compel the model to explore different “drug spaces”, as evidenced by the lowest value obtained by the Tani-Inf sampling comparatively to the other two methods.

Finally, a selection process was implemented to filter out molecules that do not have drug-like properties. Specifically, molecules that do not fall in the regions presented in Fig. 9 are excluded. The identification of the solutions that provide more adequate compromises was performed by employing the Pareto diagram, which is a method for representing the set of solutions to define the best trade-off between competing objectives.

**Fig. 11:**
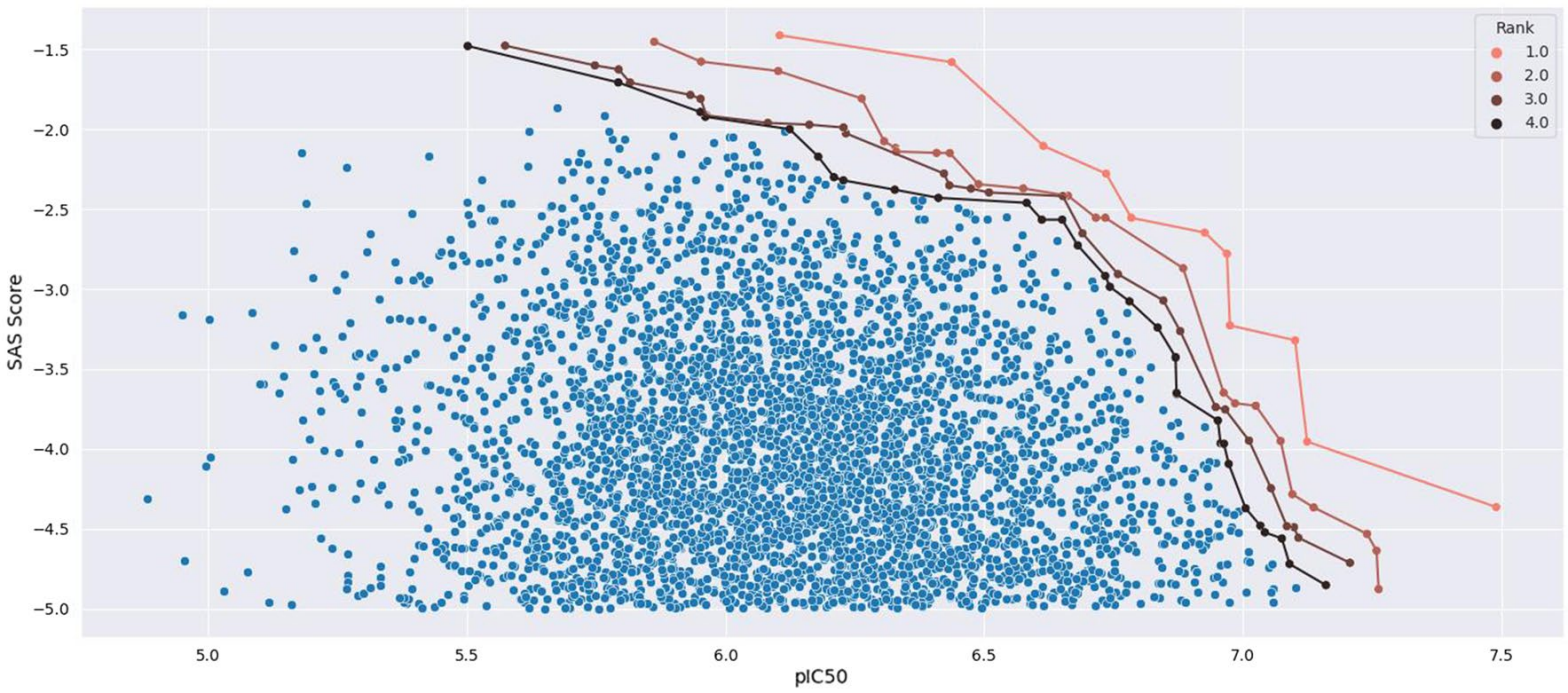
Determination of the set of selected molecules. Pareto diagram containing the approximated Pareto front in 4 layers, with the non-dominated scores of $\left( P_{IC50}(m), -SAS(m)\right)$ in red

The best of the molecules generated were selected by the non-dominated sorting algorithm based on different defined objective vectors (see Definition 1). Figure 11 shows the scatter plot for objective vector $\left( P_{IC50}(m), -SAS(m)\right)$. The objective in this case is to select the molecules that have high $P_{IC50}$ while minimizing the SAS or maximizing the negative of this value. The Additional file 1: Figures S6–S7 contains the Pareto diagrams for the two other defined score vectors.

**Fig. 12:**
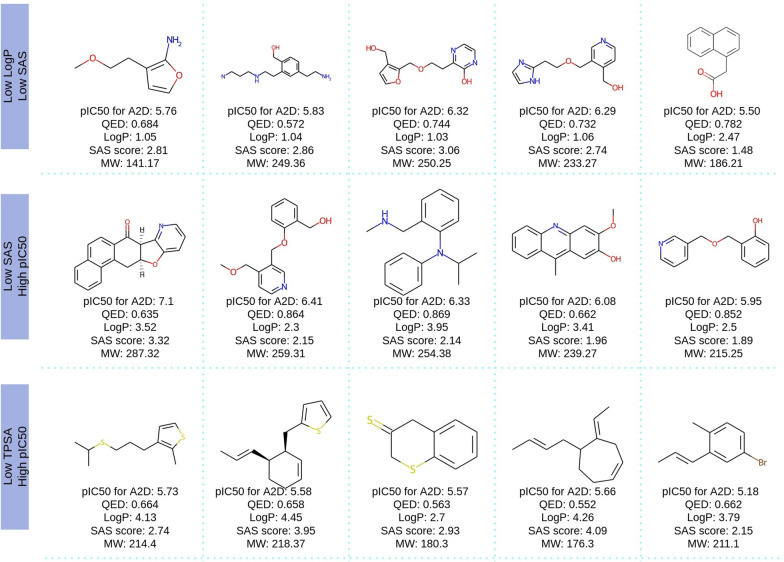
Distribution of the predicted *pIC*50 values for different sampling methods from the ADORA2A dataset

Figure 12 shows a sample of five molecules from the ranked Pareto layers of the Pareto diagrams with/ without the stereochemical information from three objective vectors defined in Definition 1. The selected molecules from the three datasets contain all the stereochemical information they need to be fully defined in their SMILES. Note that all stereocenters have enough information not to leave doubts about what stereoisomer we are focusing on. This information is all encoded in each SMILES string. Predicted pIC50 is depicted in the legend. The greater this value, the higher the binding affinity, contrary to the SA score.

**Technical notes** The implementations were in a computer with processors AMD Ryzen 9 3900X 12-core, 64 GB RAM and GPU Nvidia RTX 2070 8GB of GDDR6 VRAM using CUDA 10.1 with operating system Ubuntu 20.04.3 LTS. The results were coded in Python 3.8.3 using Tensorflow 2.3. The chemistry library used throughout is RDKit 2020_09_2 [43].

## Conclusion

We propose a GAN-based framework that aims at generating and optimizing new molecules. As GANs cannot be straightforwardly applied to discrete data, which is the case of SMILES strings, we propose the use of an Encoder–Decoder model in order to obtain continuous representation of molecules. This model reached 98.8% of correctly reconstructed SMILES strings on a hold-out test set and 99.9% on the train set, which shows an improvement when compared to the state of the art [21]. It is worth noting that our model is trained on a dataset that includes stereo-chemical information, which is often overlooked in most works due to its increased complexity but is critical in drug discovery. By passing the training data, that comprises SMILES strings, through the Encoder, an equivalent dataset is obtained that, instead of SMILES, contains fixed length continuous vectors. The representation is then used to train the GAN, more specifically a WGAN-GP. This type of model is able to implicitly learn the distribution of the training data and generate new instances following that same distribution by pairing up two competing neural networks: a Generator and Discriminator. Once the WGAN-GP is trained, the Generator network is used to generate new vectors that are then converted into SMILES strings by the Decoder network. Furthermore, we have included a multi-optimization strategy by including an extra step in the feedback loop of the proposed model to make it capable of optimizing multiple traits collectively. In particular, we applied the non-dominated sorting algorithm to select the best-generated molecules, a proven multiobjective optimization method. We showed that the trained WGAN-GP was able to replicate the distribution of the training data in terms of the predicted binding affinity for the KOR while still generating molecules with high levels of diversity (0.890 for the external diversity and 0.887 for the internal diversity). Even though the low percentage of valid generated molecules (30.2%) and the time required to train the models is clearly a drawback of this method, it is counterbalanced by the high diversity of the generated compounds and the percentage of their uniqueness (100%).

Once the WGAN-GP was trained on the dataset created by the Encoder, a feedbackGAN based optimization strategy is employed that consisted of continuing the training of the model and generating new molecules at every epoch. These new molecules are then evaluated according to their binding affinity for the KOR and the best scoring generated molecules replaced the worst scoring entries in the training data, resorting to a feedback loop. The obtained results proved that this strategy successfully resulted in shift of the generated distribution, with its mean moving from 5.984 for the unbiased model to 7.383 when aiming to maximize the predicted *pIC*50 of the generated molecules. Along with this, there was also an increase in the validity of the generated compounds from 30.2% to 62.3% with the internal diversity oscillating around 0.88 which implies that the model did not suffer from mode collapse. Interestingly, the external diversity increased as the optimization process proceeded, meaning that the framework was indeed able to generate novel compounds with binding affinities as high as 9.18. In this sense, the devised framework was effectively maximized, though it should be noted that this was accompanied by an increase in the complexity of the generated compounds proved by the synthetic accessibility scores of the generated molecules.

The overall framework has the advantage that only the optimization step needs to be adapted to different problems and goals, simply requiring for that a problem-specific scoring metric function.

## Supplementary Information

**Additional file 1.** Additional experimental analysis of the GAN architecture.

## Data Availability

All python code for this study is publicly available at https://github.com/larngroup/GAN-Drug-Generator. Contact: maryam@dei.uc.pt.

## Footnotes

1 The “Tani-Inf” and “Tani-Sup” correspond to the inferior and superior Tanimoto similarity, respectively, and not the Tanimoto distance.
