# Correction to: Designing optimized drug candidates with Generative Adversarial Network

**DOI:** 10.1186/s13321-022-00631-6

**Published:** 2022-08-11

**Authors:** Maryam Abbasi, Beatriz P. Santos, Tiago C. Pereira, Raul Sofa, Nelson R. C. Monteiro, Carlos J. V. Simões, Rui M. M. Brito, Bernardete Ribeiro, José L. Oliveira, Joel P. Arrais

**Affiliations:** 1 Department of Informatics Engineering, Univ Coimbra, Centre for Informatics and Systems of the University of Coimbra, Coimbra, Portugal; 2 BSIM Therapeutics, Instituto Pedro Nunes, 3030-199 Coimbra, Portugal; 3 IEETA, Department of Electronics, Telecommunications and Informatics, University of Aveiro, Aveiro, Portugal; 4 Department of Chemistry, Universidade de Coimbra, CQC-IMS, 3004-535 Coimbra, Portugal

## Correction to: J Cheminform 14: 40 (2022) https://doi.org/10.1186/s13321-022-00623-6

The original publication of this article [1] contained 2 errors. The incorrect and correct information is shown below, the original article has been updated.

Author name

- The incorrect author name is: Rui Brito.
- The correct author name is: Rui M. M. Brito.

Affiliation

- The affiliation “Universidade de Coimbra, CQC-IMS, Department of Chemistry, 3004-535 Coimbra, Portugal” was missing from Dr. Brito.
