# Supplementary material for: Designing optimized drug candidates with Generative Adversarial Network
Source: J Cheminform. 2022 Jun 26;14:40. doi: 10.1186/s13321-022-00623-6 (PMC9233801; doi:10.1186/s13321-022-00623-6)
Supplement: Supplementary file 1 — Additional file 1. Additional experimental analysis of the GAN architecture. [file 13321_2022_623_MOESM1_ESM.pdf]

Designing optimized drug candidates with Generative Adversarial Network: supplemental document

1. INTRODUCTION

This is a supplemental document to accompany the article "Designing optimized drug candidates with Generative Adversarial Network" in the Cheminformatics journal. This document contains an in-depth analysis of different architectures and hyperparameters for an Encoder-Decoder model, an extra minimization experiment that validates the versatility of the model, and examples of the generated molecules and corresponding drug-like properties. Moreover, it has detailed information on the multiobjective algorithm NSGA-II to select the best molecules from different objective point of view.

2. ANALYSIS OF ENCODER DECODER MODEL

This section explains in more details the experimental analysis and exhaustive grid search strategy that was employed to find the best structure and set of hyperparameters for the Encoder-Decoder model. The aim of this model is to convert the molecular compounds into continuous latent space vectors and reconstruct them correctly. It should be noted that two different structures for the Encoder input layer were considered: one with One Hot Encoding (OHE) and the other with embedding.

A. Datasets

The dataset used to do the experimental analysis on the Encoder-Decoder model in order to find the best architecture and set of parameters was the ChEMBL dataset [1].

Once the best architecture and set of hyperparameters had been defined, the model was trained on two other more complex datasets: composed_dataset_1 and composed_dataset_2 which contain 100,000 and 500,000 drug-like molecules, respectively, that were retrieved from the remaining datasets mentioned in Table S1. This resulted in datasets that include a wider variety of compounds and molecules with and without sterochemestry.

The dataset used to do experimental analysis throughout this section includes 500,000 SMILES strings of drug-like small molecules retrieved from the ChEMBL database [1] and from the dataset used by [2] which contains biogenic structures.

All the SMILES strings were canonicalized, and there were no duplicates. The SMILES strings are preprocessed by being tokenized character by character, adding 'G' as the first token of each SMILES and 'A' at the end and for padding. The SMILES are then either One-hot Encoded (OHE), where each token becomes a binary vector or passed through an embedding layer that converts each token into a dense vector that is learned by the model (Decoder).

B. Encoder-Decoder with OHE Structure

This section presents the results for the model with the structure that contains OHE layer as input to the Encoder. Table S2 shows the set of parameters that were studied in this part. The models are evaluated on their ability to correctly reconstruct 1,000 SMILES strings from the training set and 1,000 SMILES strings from the hold-out test set. This approach was chosen in order to analyse the generalization capability of the model. The closer the percentage of correctly reconstructed molecules in the test set to the one in the train set, the better the generalization of the model.

In order to independently evaluate the effect of each hyperparameter, several experiments were conducted and evaluated on an hold-out test set that contained 1,000 SMILES strings.

Table S3 shows the results obtained when keeping all the hyperparameters fixed with the exception of the number of encoder BLSTM layers which is always the same as the number of decoder LSTM layers. The number of training data was fixed at 100,000 SMILES strings, 512 units were used for the BLSTM layers (256 for each direction) and for the LSTM layers. The batch normalization momentum was set to 0.9 and the Noise standard deviation to 0.1. The model

Table S1. Summary of the datasets used throughout the experiment.

Dataset	# Compouds	Labeled	Observations
ChEMBL	1,178,946	No	-
Zinc Biogenic	108,283	No	-
ad2a	4,729	Yes	ChEMBL ID 251
KOR	5,262	Yes	ChEMBL ID 237
jak2	1,697	Yes	ChEMBL ID 2971
bbbp	1,340	Yes	-
composed_dataset_1	100,000	No	-
composed_dataset_2	500,000	No	-

Table S2. Search space for finding the optimal set of parameters of the proposed Encoder-Decoder model.

Parameters	Search Space
Number of Layers	[1,2,3]
Number of LSTM/BLSTM Units	[256,512,1024]
Batch Size	[16,32,64,128,256]
Batch Normalization Momentum	[0.7,0.8,0.9,0.95]
Latent Dimension	[64,128,512,1024]
Noise Standard Deviation	[0.1,0.15,0.2,0.25]
Training Data	[10,000, 100,000, 200,000, 500,000]

was trained using the Adam Optimizer with a batch size of 128. As it can be seen, regarding the percentage of correctly reconstructed SMILES in the train set, all models performed really well with 99.7%, 100.0% and 100.0% for 1, 2, and 3 layers, respectively. However, regarding the test set, the model with two layers clearly outperformed the remaining models by reaching 94.1% of correctly reconstructed compounds, showing its improved generalization capability. The use of two encoder BLSTM layers was chosen for the following experiments and a structure of this model is represented in Figure 2 of the article.

Table S3. Results for different number of encoder BLSTM layers and decoder LSTM layers (Encoder-Decoder with OHE structure). Dataset# =100,000 test#=1000 BLSTM/LSTM units=512 Latent dimension=512 Batch size=128 Batch normalization momentum=0.9 Optimizer=Adam Noise std=0.1

Encoder BLSTM layers#	Decoder LSTM Layers#	Last Ep.	%Correctly Reconstruc (Train)	%Correctly Reconstruc (Test)	%Valid (Train)	%Valid (Test)	Train Time (hh:mm:ss)	Total Run Time (hh:mm:ss)
1	1	48	99.7	90.5	100.0	96.2	00:35:10	02:05:03
**2**	**2**	**33**	**100.0**	**94.1**	**100.0**	**97.7**	**00:55:55**	**02:30:45**
3	3	27	100.0	91.2	100.0	97.7	01:12:11	02:48:49

Regarding the number of BLSTM/LSTM units, the results of the experiments are shown in table S4 from which it can be concluded that 512 units produces the model with better generalization

capabilities.

Table S4. Results for different number of BLSTM/LSTM units (Encoder-Decoder with OHE structure). Dataset#=100,000 test#=1000 BLSTM/LSTM layers=2 Latent dimension=512 Batch size=128 Batch normalization momentum=0.9 Optimizer=Adam Noise std=0.1

BLSTM /LSTM units	Last Ep.	Correctly Reconstruct (Train)%	Correctly Reconstruct (Test)%	Valid (Train) %	Valid (Test) %	Train Time (hh:mm:ss)	Total Run Time (hh:mm:ss)
256	38	100.0	92.9	100.0	97.5	00:40:17	02:13:37
**512**	**33**	**100.0**	**94.1**	**100.0**	**97.7**	**00:55:55**	**02:30:45**
1024	45	99.6	89.6	99.8	94.7	03:03:34	04:34:52

After setting the number of units to 512, the effect of using different batch sizes: 16, 32, 64, 128 and 256 was evaluated. According to table S5, a batch size of 64 returned the best results with 93.7% of correctly reconstructed molecules in the test set. It is interesting to note that the worst result, both in terms of train and test set, was obtained for a batch size of 16.

Table S5. Results for different number of batch size (Encoder-Decoder with OHE structure) Dataset#= 00,000 test#=1000 BLSTM/LSTM layers=2 BLSTM/LSTM units=256 Latent dimension=512 Batch normalization momentum=0.9 Optimizer=Adam Noise std=0.1

Batch Size	Last Ep.	Correctly Reconstruct (Train)%	Correctly Reconstruct (Test)%	Valid (Train) %	Valid (Test) %	Train Time (hh:mm:ss)	Total Run Time (hh:mm:ss)
16	20	94.3	86.0	98.3	96.4	01:38:46	03:12:46
32	28	99.0	92.0	99.9	97.5	01:48:40	03:20:16
**64**	**44**	**99.9**	**93.7**	**100.0**	**98.4**	**01:26:39**	**03:01:33**
128	38	100.0	92.9	100.0	97.5	00:40:17	02:13:37
256	45	99.9	92.7	100.0	96.6	00:28:44	02:00:16

The proposed structure for the Encoder-Decoder model includes a Batch Normalization Layer between every other type of layer. This type of layer requires the user to define the value for the Batch Normalization Momentum (BNM) which is the momentum of the moving average used during inference. Table S6 shows the results for four different BNM values: 0.95, 0.9, 0.8 and 0.7. The four models performed fairly similar, indicating that this parameter does not have a critical effect on the overall performance. Nevertheless, this parameter was set to 0.9 from here onwards.

The Latent Dimension defines the dimension of the context vectors. The lower this value, the more the information will have to be compressed and potentially lost. Table S7 summarizes the performance of models with different latent dimensions: 64, 128, 256, 512 and 1024. Even though the best model in terms of the percentage of correctly reconstructed molecules was the one with a latent dimension of 1024 (with 93.9%), this parameter was fixed at 256 for two reasons: the performance was similar (93.2%) and a latent dimension of 256, as opposed to 1024, would require less running time when implementing the full framework.

The last layer of the Encoder part of the model is responsible for adding Gaussian Noise to the vector so that the model can become more robust at translating between SMILES strings and context vectors. Therefore, the effect of the standard deviation of the distribution was studied and the results are shown in Table S8. The model with a standard deviation of 0.2 returned the highest performance with 92.7% of correctly reconstructed molecules in the test set.

Table S6. Results for different values Batch Normalization Momentum (BNM) (Encoder-Decoder with OHE structure). Dataset#=100,000 test#=1000 BLSTM/LSTM layers=2 BLSTM/LSTM units=256 Latent dimension=512 Batch size=128 Optimizer=Adam Noise std=0.1

Batch Normalization Momentum	Last Ep.	Correctly Reconstruct (Train)%	Correctly Reconstruct (Test)%	Valid (Train) %	Valid (Test) %	Train Time (hh:mm:ss)	Total Run Time (hh:mm:ss)
0.95	38	99.9	92.6	99.9	97.5	00:40:57	02:15:51
**0.9**	**30**	**100.0**	**92.7**	**100.0**	**96.3**	**00:32:51**	**02:06:20**
0.8	27	99.8	92.4	100.0	97.4	00:28:57	02:00:46
0.7	29	99.2	92.2	99.6	96.9	00:29:39	01:48:29

Table S7. Results for different latent dimensions (Encoder-Decoder with OHE structure). Dataset#=100,000 test#=1000 BLSTM/LSTM layers=2 BLSTM/LSTM units=512 Batch size=128 Batch normalization momentum=0.9 Optimizer=Adam Noise std=0.1

Latent Dimension	Last Ep.	Correctly Reconstruct (Train)%	Correctly Reconstruct (Test)%	Valid (Train) %	Valid (Test) %	Train Time (hh:mm:ss)	Total Run Time (hh:mm:ss)
64	41	100.0	91.5	100.0	96.6	01:08:07	02:41:29
128	35	99.9	91.9	99.9	96.0	00:58:19	02:31:33
256	35	100.0	93.2	100.0	97.4	00:58:40	02:33:03
512	30	100.0	92.7	100.0	96.3	00:32:51	02:06:20
1024	34	100.0	93.9	100.0	97.6	00:57:08	02:30:53

As a final experiment, the impact of the number of training data was also evaluated and the results are summarized in Table S9. As expected, the higher the number of training data, the higher the performance of the model. With 500,000 SMILES strings it was possible to achieve 100.0% and 99.1% of correctly reconstructed molecules in the train and test sets, respectively.

Table S8. Results for different values of Noise Standard Deviation (Encoder-Decoder with OHE structure). Dataset#=100,000 test#=1000 BLSTM/LSTM layers=2 BLSTM/LSTM units=512 Latent dimension=256 Batch size=128 Batch normalization momentum=0.9 Optimizer=Adam

Noise Std	Last Ep.	Correctly Reconstruct (Train)%	Correctly Reconstruct (Test)%	Valid (Train) %	Valid (Test) %	Train Time (hh:mm:ss)	Total Run Time (hh:mm:ss)
0.1	36	100.0	91.3	100.0	97.3	01:00:35	02:31:44
0.15	27	99.9	91.2	100.0	96.6	00:46:17	02:17:11
**0.2**	**31**	**100.0**	**92.7**	**100.0**	**98.1**	**00:52:02**	**02:26:19**
0.25	30	100.0	90.8	100.0	97.3	00:50:21	02:25:24

Table S9. Results for different number of SMILES in dataset (Encoder-Decoder with OHE structure). Test#=1000 BLSTM/LSTM layers=2 BLSTM/LSTM units=512 Latent dimension=256 Batch Size=128 Batch normalization momentum=0.9 Optimizer=Adam Noise std=0.1

SMILES Num	Last Ep.	Correctly Reconstruct (Train)%	Correctly Reconstruct (Test)%	Valid (Train) %	Valid (Test) %	Train Time (hh:mm:ss)	Total Run Time (hh:mm:ss)
10000	49	25.5	8.5	63.0	53.7	00:08:29	01:44:47
100000	34	99.8	83.4	100.0	95.2	00:56:39	02:29:42
200000	44	100.0	96.5	100.0	98.7	02:25:00	03:59:04
**500000**	**33**	**100.0**	**99.1**	**100.0**	**99.8**	**07:01:27**	**09:10:33**

C. Encoder-Decoder with Embedding Structure

As OHE is known to be a sparse and high-dimensional type of encoding [3], the use of an embedding layer as the input layer to the Encoder is studied in this section. An Embedding layer is expected to be a more computationally efficient approach and to retain information about the relations between atoms that would not be present when employing OHE.

Therefore, this section summarizes the results for the model that contains an embedding layer as input following a strategy similar to the previous section. Table S10 shows the set of parameters that are studied in this part.

Table S11 shows the results obtained for a different number of encoder BLSTM layers (which is equal to the number of decoder LSTM layers): 1, 2 and 3. From it we can conclude that, as with the previous section, a model with two layers returns the highest percentage of correctly reconstructed molecules with 100.0% and 94.3% for the train and test sets, respectively. For this experiment the number of training data was set to 100,000, the number of BLSTM/LSTM units and latent dimension were both set to 512 and the embedding dimension to 256. The models were trained with a batch size of 128 and using the Adam Optimizer. The BNM was set to 0.9 and the noise standard deviation to 0.1.

The next step was to evaluate the number of BLSTM/LSTM units. A summary of the results is shown in Table S12 where 256, 512 and 1024 units were considered. The model with 512

Table S10. Search space for finding the optimal set of parameters of the proposed Encoder-Decoder model (with Embedding structure).

Parameters	Search Space
Number of Layers	[1,2,3]
Number of BLSTM Units	[256,512,1024]
Batch Size	[16,32,64,128,256]
Embedding Dimension	[64,128,256,512]
Latent Dimension	[64,128,256,512,1024]
Training Data	[50,000, 100,000, 500,000, 1,000,000]

Table S11. Results for different number of encoder BLSTM layers and decoder LSTM layers (Encoder-Decoder with Embedding structure). Dataset#=100,000 test# =1000 BLSTM/LSTM units=512 Embedding dimension=256 Latent dimension=512 Batch size=128 Batch normalization momentum=0.9 Optimizer=Adam Noise std=0.1

Encoder BLSTM Layers #	Decoder LSTM Layers #	Last Ep.	Correctly Reconstruct (Train)%	Correctly Reconstruct (Test)%	Valid (Train) %	Valid (Test) %	Train Time (hh:mm:ss)	Total Run Time (hh:mm:ss)
1	1	23	99.9	90.5	100.0	96.9	00:21:04	01:51:14
**2**	**2**	**33**	**100.0**	**94.3**	**100.0**	**98.3**	**01:00:56**	**02:33:26**
3	3	29	100.0	91.7	100.0	98.2	01:22:05	02:58:28

BLSTM/LSTM units clearly outperformed the competing ones by reaching 100.0% and 94.3% of correctly reconstructed molecules from the train and test sets, respectively.

Table S12. Results for different number of BLSTM/LSTM units (Encoder-Decoder with Embedding structure). Dataset#=100,000 test#=1000 BLSTM/LSTM layers=2 Embedding dimension=256 Latent dimension=512 Batch size=128 Batch normalization momentum=0.9 Optimizer=Adam Noise std=0.1

BLSTM /LSTM units	Last Ep.	Correctly Reconstruct (Train)%	Correctly Reconstruct (Test)%	Valid (Train) %	Valid (Test) %	Train Time (hh:mm:ss)	Total Run Time (hh:mm:ss)
256	27	99.9	92.8	100.0	97.2	00:30:15	02:04:32
**512**	**33**	**100.0**	**94.3**	**100.0**	**98.3**	**01:00:56**	**02:33:26**
1024	32	99.7	83.0	99.8	95.3	02:28:34	04:05:02

Table S13 shows the results obtained for different batch sizes: 16, 32, 64, 128 and 256. The percentages of correctly reconstructed train and test molecules were similar for batch sizes of 64 and 128. A batch size of 64 was used from here onwards.

Regarding the choice of the embedding dimension, the results are presented in table S14. There was no outstanding model which prompted the experiment showed in Table S16 where an exhaustive comparison between the values chosen for the embedding and latent dimensions is performed. From Table S16, the model with an embedding dimension of 256 and a latent dimension of 256 was chosen due to its higher performance and better generalization capability by correctly reconstructing 95.2% of the molecules in the test set.

Lastly, an evaluation regarding the size of the training data was performed. Once again, and as

shown in Table S15, using a larger dataset results in an higher performance of the model.

Table S13. Results for different number of batch size (Encoder-Decoder with Embedding structure). Dataset#=100,000 test#=1000 BLSTM/LSTM layers=2 BLSTM/LSTM units=512 Embedding dimension=256 Latent dimension=512 Batch normalization momentum=0.9 Optimizer=Adam Noise std=0.1

Batch Size	Last Ep.	Correctly Reconstruct (Train)%	Correctly Reconstruct (Test)%	Valid (Train) %	Valid (Test) %	Train Time (hh:mm:ss)	Total Run Time (hh:mm:ss)
16	36	35.3	70.0	98.5	93.5	04:05:51	05:37:50
32	12	18.7	17.0	81.5	78.9	00:52:41	02:24:09
**64**	**35**	**100.0**	**92.2**	**100.0**	**98.5**	**01:26:57**	**03:01:01**
128	31	99.7	92.2	100.0	97.6	00:57:14	02:30:01
256	29	100.0	91.0	100.0	96.3	00:47:11	02:19:49

Table S14. Results for different number of embedding dimension in encoder (Encoder-Decoder with Embedding structure). Dataset#=100,000 test#=1000 BLSTM/LSTM layers=2 BLSTM/LSTM units=512 Latent dimension=512 Batch Size=128 Batch normalization momentum=0.9 Optimizer=Adam Noise std=0.1

Embedding Dimension	Last Ep.	Correctly Reconstruct (Train)%	Correctly Reconstruct (Test)%	Valid (Train) %	Valid (Test) %	Train Time (hh:mm:ss)	Total Run Time (hh:mm:ss)
64	39	100.0	94.5	100.0	97.9	01:11:27	02:44:08
128	31	100.0	94.2	100.0	98.1	00:57:22	02:22:57
256	31	99.7	92.2	100.0	97.6	00:57:14	02:30:01
512	30	99.9	94.6	99.9	98.2	00:55:26	02:28:16

Table S15. Results for different number of SMILES in dataset (Encoder-Decoder with Embedding structure). Test#=1000 BLSTM/LSTM layers=2 BLSTM/LSTM units=512 Embedding dimension=256 Latent dimension=512 Batch Size=128 Batch normalization momentum=0.9 Optimizer=Adam Noise std=0.1

# SMILES	Last Ep.	Correctly Reconstruct (Train)%	Correctly Reconstruct (Test)%	Valid (Train) %	Valid (Test) %	Train Time (hh:mm:ss)	Total Run Time (hh:mm:ss)
50000	38	100.0	84.5	100.0	93.8	00:15:22	00:49:55
100000	37	99.9	94.5	99.9	98.0	00:29:44	01:04:28
**500000**	**21**	**99.9**	**98.8**	**100.0**	**99.8**	**01:26:54**	**02:01:49**

D. The Proposed Encoder-Decoder Model

Taking in consideration the previous results, the chosen encoder-decoder model contains two bidirectional LSTM layers with 512 units each, 256 for each direction. Both the embedding dimension and latent dimension were set to 256. All the batch normalization layers had a batch normalization momentum of 0.9. The Gaussian noise layer added noise with a standard deviation of 0.1. The model was trained using the Adam optimizer with a learning rate of 0.01, a batch size

Table S16. Results for comparison between embedding dimension and latent dimension (Encoder-Decoder with Embedding structure). Dataset#=100,000 test#=1000 BLSTM/LSTM layers=2 BLSTM/LSTM units=512 Batch size=128 Batch normalization momentum=0.9 Optimizer=Adam Noise std=0.1

Embedding Dimension	Latent Dimension	Last Ep.	Correctly Reconstruct (Train)%	Correctly Reconstruct (Test)%	Valid (Train) %	Valid (Test) %	Train Time (hh:mm:ss)	Total Run Time (hh:mm:ss)
64	64	34	100.0	92.1	100.0	97.1	00:27:48	01:02:32
64	128	32	100.0	92.3	100.0	97.3	00:26:15	01:01:07
64	256	33	100.0	93.7	100.0	98.0	00:27:04	01:01:59
64	512	25	99.9	91.7	100.0	97.1	00:20:44	00:55:37
64	1024	27	99.7	92.9	99.9	97.3	00:22:23	00:57:13
128	64	50	100.0	93.2	100.0	97.4	00:40:32	01:15:26
128	128	46	100.0	92.0	100.0	96.7	00:37:25	01:12:13
128	256	25	99.9	90.7	100.0	98.0	00:20:44	00:55:38
128	512	33	100.0	92.6	100.0	96.5	00:27:08	01:02:04
128	1024	33	100.0	92.1	100.0	97.4	00:27:06	01:01:55
256	64	35	100.0	91.2	100.0	97.7	00:28:39	01:03:32
256	128	35	100.0	93.2	100.0	97.6	00:28:31	01:03:21
**256**	**256**	**39**	**99.9**	**95.2**	**100.0**	**97.8**	**00:31:54**	**01:06:47**
256	512	37	100.0	93.3	100.0	98.3	00:30:26	01:05:14
256	1024	26	99.9	94.1	100.0	97.9	00:21:24	00:56:20
512	64	38	99.9	91.3	99.9	96.6	00:31:01	01:05:57
512	128	44	100.0	92.7	100.0	97.5	00:35:48	01:10:40
512	256	36	100.0	90.7	100.0	96.6	00:29:28	01:04:25
512	512	27	100.0	92.2	100.0	96.7	00:22:21	00:57:28
512	1024	31	100.0	93.5	100.0	97.6	00:25:33	01:00:35
1024	64	34	100.0	92.6	100.0	96.8	00:27:56	01:03:00
1024	128	29	99.9	93.8	100.0	98.0	00:24:00	00:59:00
1024	256	30	99.8	92.1	99.8	97.3	00:24:41	00:59:40
1024	512	31	100.0	93.4	100.0	98.2	00:25:30	01:00:34
1024	1024	32	100.0	93.6	100.0	97.2	00:26:16	01:01:21

of 128, and the total number of epochs was set to 100, but only the best models regarding the validation loss were kept (10% of the training data was set as validation data).

This model was trained using the “composed_dataset_1" and “composed_dataset_2" (see Section A on Page 1 for more details). The results from training with these datasets which are more complex due to including a wider range of molecules and also stereo-chemical information are summarized in Table S17.

In both cases, the model reaches high percentages of correctly reconstructed molecules for the train and test sets. As expected, the model trained with 500,000 molecules learned to generalize better (with 99.2% and 99.0% for the train and test set, respectively) when compared to the one trained on only 100,000. It should be noted that molecules that are correctly reconstructed are automatically valid. Validity (evaluated by RDkit [4]) is constantly higher than the percentage of correct reconstruction, which means that some molecules are reconstructed into valid molecules but not the intended ones.

Dataset	#Training Data	%Correct R. (train set)	%Correct R. (test set)	%Validity (train set)	%Validity (test set)
composed_dataset_1	100,000	98.6	96.8	99.7	99.1
**composed_dataset_2**	**500,000**	**99.2**	**99.0**	**99.9**	**99.8**

Table S17. Performance of the Encoder-Decoder model for 100,000 and 500,000 training data.

3. ANALYSIS OF OPTIMIZATION WITH FEEDBACKGAN:MINIMIZING KOR AFFINITY

To evaluate the versatility of the framework and also to study possible off-target effects, the unbiased WGAN-GP model was optimized for 500 epochs with the goal of shifting the probability distribution towards the minimization of the predicted $pIC50$. Figure S1 shows the distribution of predicted $pIC50$ values in terms of a probability density for intervals of 100 epochs while Table S18 presents more detailed information regarding the distributions of predicted $pIC50$, diversity, uniqueness and validity of the sampled molecules for every 50 epochs. From this information, it is possible to conclude that there was a first shift towards higher predicted $pIC50$ values at the beginning of the optimization process before it started to move towards the goal of minimization. This can be attributed to the fact that, as previously explained, before starting the optimization process we reduced the training dataset from 100,000 to 5,000 compounds, effectively breaking the flow of training. We also believe that this is the reason for the decrease in validity from 30.2% (unbiased) to 15.2% (epoch 50) which is then followed by a constant increase as the model adapts to the new training data.

The internal and external diversity remain high throughout the optimization, reaching the highest values for the 500th epoch with 0.904 and 0.906, respectively. This implies that this optimization framework is capable of generating new valid molecules while maintaining high values of uniqueness, which is constantly close to 100.0%.

The overall shift between the unbiased and the optimized model (500 epochs) is significantly less than the one observed for the maximization experiment This can be largely attributed to the fact that the unbiased distribution is positively skewed which makes it less likely to sample a high number of molecules with low $pIC50$ therefore slowing down the optimization process. In this sense, to achieve better optimization results, one would either have to train the model for a higher number of epochs or sample more molecules at each optimization epoch.

Table S18. Comparison of $pIC50$ distribution measures throughout the optimization process (minimization of KOR affinity).

	Unbiased	50 epoch	100 epoch	150 epoch	200 epoch	250 epoch	300 epoch	350 epoch	400 epoch	450 epoch	500 epoch
Max pIC50	8.454	8.611	8.201	8.110	8.202	7.990	8.025	8.110	7.591	7.603	7.837
Mean pIC50	5.984	6.485	6.265	6.177	6.110	6.013	5.925	5.894	5.830	5.814	**5.737**
Min pIC50	5.083	5.133	5.141	5.140	5.091	5.052	4.971	4.995	5.058	5.030	5.001
Int Div	0.887	0.884	0.887	0.886	0.897	0.893	0.896	0.900	0.903	0.897	**0.904**
Ext Div	0.890	0.894	0.896	0.893	0.899	0.897	0.900	0.901	0.905	0.901	**0.906**
% Unique	100.0	100.0	100.0	100.0	99.9	99.9	99.7	99.9	99.7	99.7	99.2
% Valid	30.2	15.2	25.5	27.8	32.1	35.5	38.2	39.1	38.1	39.3	**41.12**

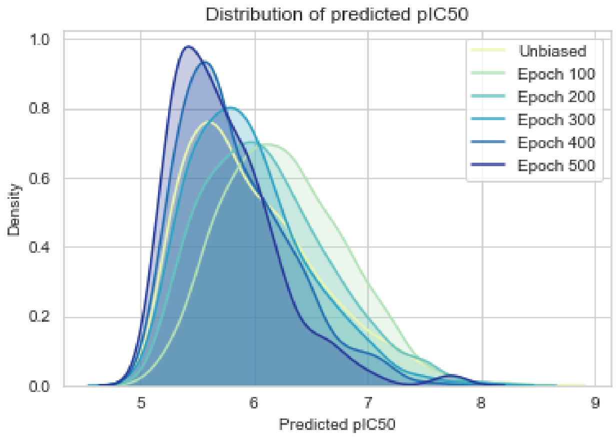

Fig. S1. Distribution of the predicted $pIC50$ values for the unbiased model and the biased model at every 100 epochs (minimization of KOR affinity).

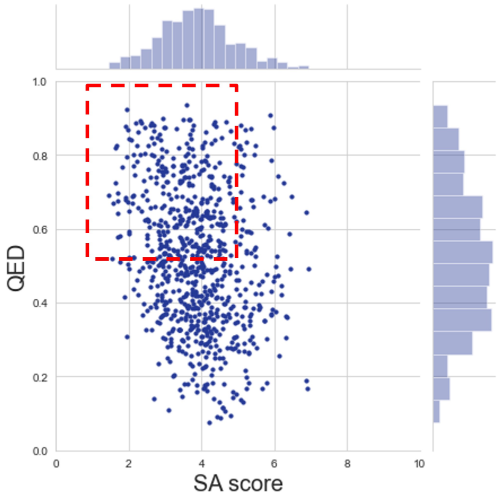

Fig. S2. Evaluation of the QED and SA score for the biased model (minimization) at 500 epochs.

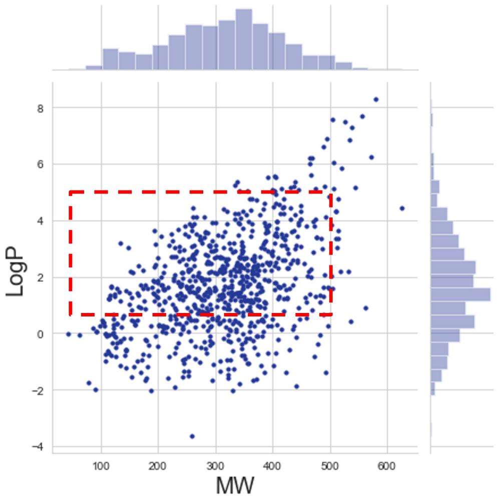

Fig. S3. Evaluation of the log P and MW for the biased model (minimization) at 500 epochs.

4. MULTIOBJECTIVE OPTIMIZATION STRATEGY

Research in multiobjective optimization problems has turned from finding a single best solution to finding a set of nondominated or Pareto optimal front. The main goal is to find solutions (molecules) that are not worse than any other solution and strictly better in at least one of the objectives (properties).

A multiobjective optimization problem (MOP) is the problem of finding values for a set of decision variables that optimize a vector objective function given a set of constraints. The components of this function form a mathematical description of performance criteria which are usually in conflict with each other. Hence, the term "optimize" means finding a solution that would be as good as possible to all objectives. A general MOP can mathematically be defined as follows:

$$\begin{aligned} \text{“maximize” } z = f(x) = \big(f_1(x), f_2(x), \ldots, f_q(x)\big) \\ s.t\ x \in X \end{aligned} \tag{S1}$$

where x is an n-dimensional decision vector, or solution, and X is the set of all feasible solutions.

Note that if some objective function is to be maximized, it is equivalent to maximize its negative. Without loss of generality, we assume *maximization* of all objectives.

The main goal of solving MOPs in terms of Pareto optimality is to find solutions that are not worse than any other solution and strictly better in at least one of the objectives. To understand how the corresponding objective vectors can be ordered, the following binary relations in $\mathbb{R}^q$ are introduced [5]. Let u and v be vectors in $\mathbb{R}^q$.

- $u \succeq v \iff u_i \geq v_i, i = 1, \ldots, q;$
- $u \succ v \iff u \neq v$ and $u_i \geq v_i, i = 1, \ldots, q;$
- $u > v \iff u_i > v_i, i = 1, \ldots, q.$

In the context of optimization, we denote the relation between objective function vectors of two feasible solutions x and x' as follows:

- if $f(x) \succeq f(x')$, then $f(x)$ weakly dominates $f(x')$.
- if $f(x) \succ f(x')$, then $f(x)$ dominates $f(x')$;
- if $f(x) > f(x')$, then $f(x)$ strictly dominates $f(x')$;

Figure S4 illustrates the concept of dominance in the biobjective case, assuming maximization. In this example, the point B strictly dominates point A. Point C strictly dominates points A and B_1. Point B_1 and B_2 are mutually non-dominated, but they are (weakly) dominated by point B.

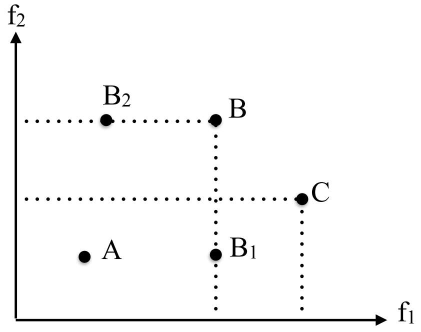

Fig. S4. A schematic illustration of the "dominance relation", assuming two maximizing objectives

The Pareto optimal set of solutions consists of all those that it is impossible to improve in any objective without a simultaneous worsening in some other objective.

Non-dominated Sorting Genetic Algorithm (NSGA-II) NSGA-II ranks the individuals in the population into layers based on dominance relation. Any two individuals in the same layer are non-dominated. An individual belongs to a certain layer if it is not dominated by any individual of the inferior layer. The first layer is computed by finding the individuals that are not dominated by any other individuals in a population. The next layers are computed similarly by ignoring the individuals that belong to the previous layers. Figure S5 (left) shows an illustrative example of the ranking in NSGA-II. The first layer $\mathcal{L}_1$ is shown with white circles. By putting aside them, the second non-dominated layer $\mathcal{L}_2$ is shown with gray circles and the last individual create the last layer (black circle). Efficient algorithms for the ranking [6].

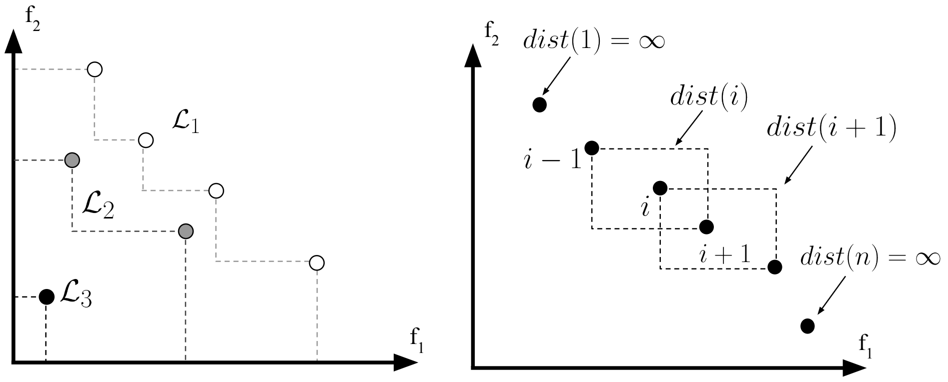

Fig. S5. Illustration of ranking (left) and crowding distance (right) in NSGA-II

In the following, we describe how NSGA-II works. Initially, a randomly generated population P_0 of N elements is created. The population is sorted into layers based on the fast non-dominated sorting step. Since the maximization of the objectives is considered, each individual is assigned with a fitness equal to the reciprocal of the non-dominated layer it belongs to (the best layer is 1). To create an offspring population Q_0, binary tournament selection, crossover and mutation operators are applied. After the first generation is created, the procedure will keep the N best individuals (elitism strategy). Algorithm S1 shows the main loop of NSGA-II at iteration t. Non-dominated layers $\mathcal{L}_1, \mathcal{L}_2, \ldots, \mathcal{L}_R$ are identified in the combined population $P_t \cup Q_t$. The next population P_{t+1} is filled starting with individuals from $\mathcal{L}_1$, then $\mathcal{L}_2$, and so on as follows. Let k be the index of a non-dominated layer $\mathcal{L}_k$ such that $|\mathcal{L}_1 \cup \mathcal{L}_2 \cup \ldots \cup \mathcal{L}_k| \leq N$ and $|\mathcal{L}_1 \cup \mathcal{L}_2 \cup \ldots \cup \mathcal{L}_k \cup \mathcal{L}_{k+1}| > N$. First, all non-dominated individuals in the levels $\mathcal{L}_1, \mathcal{L}_2, \ldots, \mathcal{L}_k$ are copied to P_{t+1}, and then the $N - |P_{t+1}|$ with the least crowded distance from $\mathcal{L}_{k+1}$ are added to P_{t+1}. This approach ensures that all non-dominated individuals from $\mathcal{L}_1$ are included in the next population if $|\mathcal{L}_1| \leq N$, and, otherwise, the selection based on a crowding distance is used to keep diversity. Finally, population Q_{t+1} is obtained from P_{t+1} by the application of selection, crossover and mutation.

Algorithm S1. The main loop of NSGA-II algorithm

```
procedure MAIN ITERATION:
    Generate layers L_1, ..., L_R from P_t ∪ Q_t with fast non-dominated sorting
    i = 1
    P = P_{t+1} = ∅
    while |P| < N do
        CrowdingDistanceCalculation(L_i)
        P = P ∪ L_i
        i = i + 1
    Sort P in non-decreasing order of crowding distance
    P_{t+1} contains the best N individuals from P
    Generate Q_{t+1} from P_{t+1} by evolutionary operators
    t = t + 1
```

Figure S6 highest scoring molecules for the minimization experiment.

Algorithm S2. The algorithm for calculating the crowding distance in NSGA-II

procedure CROWDING DISTANCE CALCULATION($\mathcal{L}$)
  $n_{\mathcal{L}} = |\mathcal{L}|$
  for each $i \in \mathcal{L}$ **do**
    $dist(i) = 0$
  for $k = 1$ *to* q **do**
    Sort individuals in layer $\mathcal{L}$ in non-increasing order of objective k
    $dist(1) = dist(n_{\mathcal{L}}) = \infty$
    for $i = 2$ *to* $n - 1$ **do**
      $dist(i) = dist(i) + f^{\mathcal{L}}_{k,i+1} - f^{\mathcal{L}}_{k,i-1}$

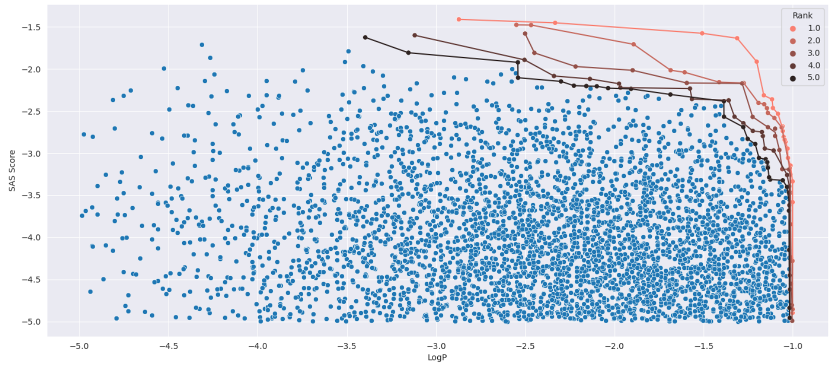

Fig. S6. Determination of the set of selected molecules. Pareto diagram containing the approximated Pareto front in 4 layers, with the non-dominated scores of $(-LogP(m), -SAS(m))$ in red.

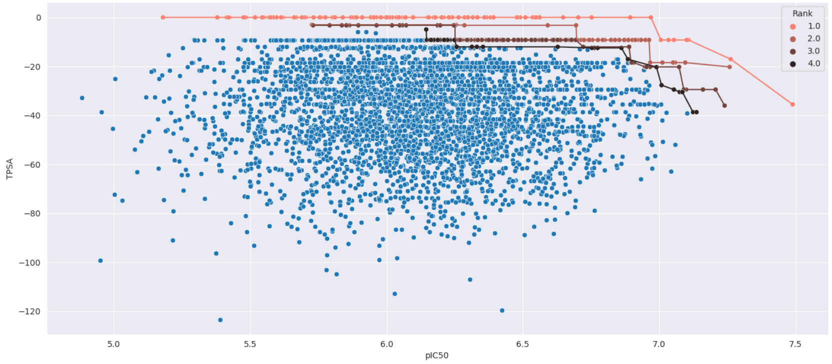

Fig. S7. Determination of the set of selected molecules. Pareto diagram containing the approximated Pareto front in 4 layers, with the non-dominated scores of $(P_{IC50}(m), -TPSA(m))$ in red.

REFERENCES

1. A. Gaulton, L. J. Bellis, A. P. Bento, J. Chambers, M. Davies, A. Hersey, Y. Light, S. McGlinchey, D. Michalovich, B. Al Lazikani *et al.*, "Chembl a large scale bioactivity database for drug discovery," Nucleic acids research **40**, 1100–1107 (2012).
2. S. Zheng, X. Yan, Q. Gu, Y. Yang, Y. Du, Y. Lu, and J. Xu, "Qbmg: quasi biogenic molecule generator with deep recurrent neural network," Journal of cheminformatics **11**, 1–12 (2019).
3. A. L. Beam, B. Kompa, A. Schmaltz, I. Fried, G. Weber, N. Palmer, X. Shi, T. Cai, and I. S.

Kohane, "Clinical concept embeddings learned from massive sources of multimodal medical data," in *PACIFIC SYMPOSIUM ON BIOCOMPUTING 2020,* (World Scientific, 2019), pp. 295–306.

4. G. Landrum *et al.*, "Rdkit: Open-source cheminformatics," (2006).
5. M. Ehrgott, *Multicriteria Optimization* (Springer, Berlin, Heidelberg, 2005).
6. M. Jensen, "Reducing the run-time complexity of multiobjective eas: The nsga-ii and other algorithms," IEEE Transactions on Evolutionary Computation **7**, 503–515 (2003).
